# Comparison of *Peganum harmala* L. leaves extract nanoformulations against herpes simplex virus type 1 guided by network pharmacology analysis

**DOI:** 10.1038/s41598-025-24155-9

**Published:** 2025-11-18

**Authors:** Basant A. Abou-Taleb, Aya M. Elbanan, Hala M. Hammoda, Ibrahim A. Abdelwahab, Mohamed M. Mohyeldin, Dina S. Ghallab

**Affiliations:** 1 Department of Pharmaceutics & Pharmaceutical Technology, Faculty of Pharmacy, Pharos University in Alexandria, Canal El Mahmoudia Street, Beside, Green Plaza Complex, Alexandria, 21648 Egypt; 2 Department of Pharmaceutics and Pharmacy practices, Alexandria University Hospitals, Alexandria University, Alexandria, Egypt; 3 Department of Pharmacognosy, Faculty of Pharmacy, Alexandria University, Alexandria, 21521 Egypt; 4 Department of Microbiology and Immunology, Faculty of Pharmacy, Pharos University in Alexandria, Canal El Mahmoudia Street, Beside, Green Plaza Complex, Alexandria, 21648 Egypt

**Keywords:** Antiviral activity, Chitosan nanoparticles, Chitosan–zinc oxide nanoparticles, Network pharmacology, Peganum harmala, TPP-Chitosan–zinc oxide nanoparticles, Zinc oxide nanoparticles, Computational biology and bioinformatics, Microbiology, Plant sciences, Drug discovery, Drug delivery, Pharmaceutics

## Abstract

Herpes simplex virus type 1 (HSV-1) is a highly prevalent viral infection with limited medications. Thus, search for safe and effective alternative treatments is urgently needed. *Peganum harmala* L. (*P. harmala*) praised with antiviral potential may afford a decent option against HSV-1. This study creatively integrated network pharmacology and nanoscience to objectively disclose the efficacy mechanism of *P. harmala* bioactive compounds and augment the antiviral potential of *P. harmala* against HSV-1 via nanotechnology. Network pharmacology analysis revealed MAPK 1, SRC, EGFR and JAK1 as the top putative HSV-1 genes highly enriched in MAPK, PI3K-Akt, and JAK-STAT signalling pathways and primarily associated with the efficacy mechanism of *P. harmala* bioactive compounds against HSV-1. Complementarily, four *P. harmala* nano-formulations were established, monitored using different pharmaceutical scores, and assessed against HSV-1 using plaque reduction assay. Experimentally speaking, *P. harmala*-CS-ZnO NPs showed higher zeta (+ 40.8) with particle-size (73.06 nm), higher entrapment (81.7%) with loading-capacity (6.8%), sustained release reaching 50.5% after 24 h and demonstrated the most promising observation against HSV-1, with viral inhibition of 54.1% which is double the effect of crude extract alone with acceptable cytotoxicity (CC_50_ = 271.4 µg/ml). This enhanced effect is possibly due to the synergistic antiviral properties of *P. harmala* bioactive compounds, chitosan, and zinc oxide nanoparticles. This complex between the ingredients chemically detected by FT-IR analysis also improved stability, cellular uptake, viral inhibition, and bioavailability. Our findings offer a solid basis for more extensive and rational clinical integration of *P. harmala* in the pharmaceutical industry to rectify human herpes viruses.

**Supplementary Information:**

The online version contains supplementary material available at 10.1038/s41598-025-24155-9.

## Introduction

Herpes simplex virus (HSV) belonging to the alpha subfamily of human herpes viruses encompasses HSV-1 and HSV-2, which are directly implicated in pandemics of varying herpes disorders^1^. Both pathogens share structural similarities and are of global concern not only because their long-lasting effects of oral or genital ulcerative lesions but also the possibility of blindness from ocular herpes and increased mortality rates from neonatal herpes or encephalitis^2^. In the fight against such quickly emerged viruses, over ten HSV prophylactic vaccinations, primarily HSV-1 vaccines, have been developed and incorporated in human clinical trials^3^. Although the vaccines could lower the risk of infection and the number of symptomatic cases among infected individuals. Nonetheless, concerns regarding the duration of protection provided by vaccines are upraised because HSV is well-equipped with various virulence factors posing a hurdle to the vaccines effectiveness^3^. Further, currently, most current medications prescribed for HSV are supportive with no definite therapy yet^3,4^. Given this widespread circumstance, search for safer and more effective alternative treatments to cope with these crippling pathogens is urgently needed. Undoubtedly, medicinal plants with plentiful biodiversity, unique molecular scaffolds and potentiating biological attributes may afford decent alternative entities against HSV^5,6^.

*Peganum harmala* L. (Zygophyllaceae), commonly called as “Harmal” or “Suryin Rue, is a perennial, herbaceous, wild flowering plant with short creeping roots and widely distributed throughout the Mediterranean’s warm temperate to subtropical regions^7^. *P. harmala* is widely utilized in traditional Chinese medicine as a stimulant for central nervous system and for or the apoplexy and lumbago treatment^8^. Further, *P. harmala* has been long used for the treatment of diabetes, hypertension, and arthritis in different folkloric cultures^9^. Form a chemical viewpoint, *P. harmala* serves as a prime store of structurally variable compounds spanning varying chemical classes including alkaloids (quinazoline alkaloids and *β*-carboline), phenolics, carotenoids, flavonoids, triterpenoids, lignans and essential oil components which positively contribute to its health-promoting impacts^10,11^ exemplified in anticancer, antidiabetic, anti-asthmatic, antiviral, anti-inflammatory, anti-hypertensive and neuroprotective properties^10,12,13^. Overviewing earlier studies regarding the antiviral activity of *P. harmala*, it was observed a previous report demonstrating the potential antiviral activity of *P. harmala* seeds extract against influenza A virus via suppression of viral RNA transcription and viral polymerase action^14^. Another leading investigation revealed the remarkable anti-HSV-2 activity of *P. harmala* extract and its derived alkaloid; harmine exerting their virucidal actions during the entry of viruses and the virions release and subsequently enhance genital herpes treatment, particularly for patients with compromised immune systems^15^. Despite these promising studies, the overall efficacy mechanism of *Harmala* bioactive compounds against HSV-1 remains vague and warrants further investigation.

As evidence-based medicine becomes more prevalent, the development of a fire-new strategy known as network pharmacology has been surfaced offering a tangible biological language to effectively screen the pharmacological actions beyond drug treatment from multiple scale perspective via creating an interacting network between bioactive compounds, disease targets and relating pathways^16,17^. Incorporating such interdisciplinary approach which aligns with the features of multi-component and multi-target effects of traditional Chinese medicine (TCM) can systemically profile the active components, and overall action mechanisms of herbal medicines in a deeper scientific scope^17,18^.

Although *P. harmala* has shown promising antiviral properties against HSV-1, challenges related to its bioavailability and targeted delivery limits its clinical application. For this reason, recent advances in nanotechnology offer innovative solutions to enhance the antiviral efficacy of *P. harmala* leaves extract through improved stability, controlled release, and targeted delivery systems. Metal oxide nanoparticles exhibit significant physical, chemical, and biological characteristics, making them an attractive resource for multiple uses^19^. The US Food and Drug Administration (FDA) has classified zinc oxide (ZnO) as a nontoxic substance^20^. Consequently, zinc oxide nanoparticles (ZnO NPs) are permitted for use in several biomedical applications. ZnO NPs are distinguished by their little toxicity and cost efficiency, making them applicable in various therapeutic areas, including wound healing, drug administration, and for anticancer, antidiabetic, antibacterial, and anti-inflammatory applications^21^. ZnO NPs are more biocompatible with human cells than zinc metal and are readily absorbed by biological tissues when compared to other nanoparticle types. Furthermore, ZnO NPs exhibit antiviral action against a wide range of viruses, including SARS-CoV-2 and other respiratory and herpes viruses such as Respiratory Syncytial Virus (RSV), Herpes Simplex Virus (HSV)^22^. ZnO NPs have antiviral activity through a number of mechanisms, including blocking viral entrance, replication, and organ spread, which can ultimately result in reactive oxygen species, oxidative damage, and viral death^22^. Compounds containing zinc demonstrated antiviral action against a variety of viruses by a variety of physicochemical mechanisms, including adhesion to the virus, blockage of virus infection, and uncoating. Additionally, these substances exhibited biological modes of action, including the inhibition of protease enzymes and viral polymerases^23^. Natural polysaccharides as cellulous, alginate, hyaluronic acid, starch, and chitosan can coat NPs to increase their antimicrobial action and decrease their toxicity, according to certain reports^24–27^. Among organic polymers, chitosan (CS) seems to be a good choice for some reasons. Firstly, it is considered as a good candidate to create nano-hybrids due to the presence of functional groups for the attachment of metal ions^28^. Moreover, (CS) is known as biocompatible, biodegradable and non-toxic polysaccharide^29,30^. Furthermore, several investigations have shown that CS and its derivatives have antibacterial properties against strains of bacteria, fungi, and viruses^29–31^. Although the exact mechanisms of chitosan against viruses have not been fully understood, several factors have been proposed for its inhibitory activity against viruses^32^.

Chitosan nanoparticles (CS NPs) have demonstrated antibacterial, biocompatible, bioactive, and non-toxic qualities. Because of their small size and positive charge, CS NPs exhibit intriguing interface and surface phenomena. According to several studies, CS NPs may be a novel treatment option for viral infections^33^. Last but not least, these CS NPs’ small sizes enhance the effects of the extracts’ active ingredients by permitting increased diffusion and penetration across the cell membrane^34^.

To allay these concerns, the current research strategy creatively integrated network pharmacology, nanoscience, and experimental validation to objectively disclose the efficacy mechanism of the bioactive compounds prevalently existing in *P. harmala* leaves extract against HSV-1 from a wholistic view. With the main scope to enhances antiviral effect of *P. harmala* leaves extract, different nano-formulations based on Zinc oxide and chitosan were established and experimentally assessed through a series of physicochemical characterization, in vitro pharmaceutical scores and antiviral effect using plaque reduction assay. The results achieved herein will provide a solid basis for a more extensive and rational clinical integration of *P. harmala* leaves extract in the pharmaceutical industry for the development of safer and more effective nutraceuticals to rectify human herpes viruses. As far as authors known, the current study offers the first integrative attempt featuring network pharmacology, nanotechnology, and experimental validation to provide an overview of the efficacy mechanism of Harmala bioactive compounds against HSV-1. Following that, nanotechnology, supported by experimental verification, is a promising strategy for reinforcing the anti-HSV-1 efficacies of *P. harmala* bioactive compounds.

## Materials and methods

### Reagents and chemicals

LC-MS grade solvents of high purity (99.9%) including methanol, acetonitrile and formic acid were obtained from Fisher Scientific, UK.

Regarding nano-formulations preparation, the main reagents were acquired as follow; the chitosan (CS) (molecular weight = 200 kDa, degree of acetylation > 90%) was supplied by alpha Chemika, an Indian company. Loba-Chemie, India, was the source of sodium-tri-polyphosphate (TPP). Tween 80 was supplied by El-Nasr Ph. Chemicals Co. in Egypt. Zinc acetate dehydrate and Sodium hydroxide (NaOH) (98%) were provided by Sigma-Aldrich Co. (USA). Analytical-grade reagents and solvents were employed.

### Plant material

#### Sample collection and extraction

*P. harmala* leaves were freshly harvested from a certified organic farm situated in Nafusa Mountain, Libya in March 2022 and taxonomically verified by a herbal consultant on Plant Taxonomy (https://powo.science.kew.org/taxon/urn:lsid:ipni.org:names:77251931-1). A voucher sample (PD-PH-006) was put down at the Pharmacognosy Department, Faculty of Pharmacy, Alexandria University. After that, the freshly collected leaves were spread out on muslin cloth, shade-dried for ten days and finely ground for extraction. In compliance with our earlier extraction protocols^35,36^, *P. harmala* leaves extract was prepared as follows: 500 g of dried powder were twice extracted in 70% ethanol for 7 days with occasional stirring and shaking followed by ultrasonication using bath apparatus (3 L Alpha Plus, Japan) at 40 °C for 60 min. Following the filtration process, the filtrates was combined, vacuum-concentrated at 40–50 °C using a rotary evaporator, lyophilized (Christ Alpha, USA), and stored at − 20 °C until later analysis.

#### UPLC-MS/MS analysis of *P. harmala* leaves extract and data processing

Under the guidance of the chromatographic protocol previously described in our previous investigations^37,38^, the chemical composition of *P. harmala* leaves extract was established using UPLC XEVO TQD triple quadruple instrument Waters Corporation, Milford, MA01757 U.S.A.

A detailed description for chromatographic analysis protocol, parameters for electrospray ionization (ESI) interface and mass data pre-processing was clarified in the supplementary material.

#### Network pharmacology study

In the second series of experiments, the annotated compounds retrieved from UPLC-MS/MS analysis of *P. harmala* leaves extract were set as the candidate compounds for network pharmacology analysis.

Undoubtedly, the two most crucial pharmacokinetic indices of a medication’s absorption, distribution, metabolism, and excretion (ADME) features are oral bioavailability (OB) and drug likeness. Accordingly, through the use of Qikprop software (Schrödinger suite 2017 A), the annotated compounds were virtually filtered out based on their oral bioavailability (OB) and Lipinski’s rule of five indices. The following criteria are part of Lipinski’s Rule (LR), which is used to identify active compounds: molecular weight (MW) ≤ 500, chemical composition with fewer than five hydrogen bond donors (Hdon ≤ 5), octanol-water partition coefficient less than five (LogP ≤ 5), and no more than ten hydrogen bond acceptors (Hacc ≤ 10)^16^. Herein, the compounds with favorable OB score ≥ 30% as well as met at least three criteria from Lipinski’s rule of five were selected.

Following that, the SMILES formats of the filtered identified compounds were employed as inputs to pinpoint the target genes in referenced genomic databases. STITCH (http://stitch.embl.de/, ver. 5.0) and Swiss Target Prediction Servers (http://www.swisstargetprediction.ch/) with the species assigned as “*Homo sapiens*” were utilized to filter out the molecular targets respecting to these candidate compounds. On the meantime, the genomic data relating to herpes viruses was retrieved from Therapeutic Target Database (TTD) and Gene Cards databases specifying the term “Herpes simplex virus”. After combining the search results and eliminating duplicate entries, a consolidated list of genes linked to herpes was gained. Further, STRING database was used to create the protein–protein interaction (PPI) network of overlapping hub targets. Next, the hub genes were loaded into the DAVID Bioinformatics platform (https://david.ncifcrf. gov) to profile the biological function and their related pathways using the Kyoto Encyclopedia of Genes and Genomes (KEGG) enrichment analysis^39,40^ selecting items with a screening criterion of *p*-value ≤ 0.05 where a bubble plot of the top 15 KEGG analysis items was created.

Lastly, the software Cytoscape (version 3.7.0) was used to create and visually analyse the compound-target-pathway network. The significance of nodes in each network was explicitly assessed using degree values, betweenness centrality, and closeness centrality impeded in a plug-in for network analysis.

#### Molecular docking studies

In order to supplement and validate the network pharmacology findings, the binding patterns between the highly correlated gene (MAPK 1) and the top efficacy compounds in Harmala leaves extract (harmine, peganone 2, and vasicine) were clarified through molecular docking analysis using Schrodinger Maestro 11.8 software (LLC, New York, NY). Accordingly, the highest-resolution X-ray crystal structure of Mitogen-activated protein kinase 1 (MAPK 1) (PBD ID 5EKO) was obtained from the Protein Data Bank (http://www.rcsb.org/pdb) where protein preparation wizard module in the Schrodinger package was then used to prepare and optimize the imported protein following our previously optimized protocol^41^ which briefly includes assignment of hydrogen bonds and bond order at pH 7.0, elimination of water molecules larger than 5 Å from the protein binding pocket, and energy minimization with a root mean square displacement (RMSD) value of 0.3 Å. The docking binding pocket was determined using the grid preparation tool with a size adjustment of ≤ 20 Å. The prepared compounds with modified chirality, ring conformations, stereochemistry, and ionization states subjected to molecular docking analysis was performed using the Glide 11.8 module (Glide, version 11.8, 2018, Schrödinger, USA), where the G score was established as a predictive scoring function of binding affinity and multiple interactions.

### Preparation of the nano-formulations

#### Preparation of *P. harmala* leaves extract-loaded Chitosan nanoparticles (*P. harmala*-CS-NPs)

According to the previously mentioned procedure, *P. harmala leaves extract*-loaded Chitosan NPs *(P. harmala* CS-NPs) were made using chitosan ionic-gelation with (TPP)^42–47^. 2 mg/ml chitosan solution was prepared; by dissolving chitosan (CS) in a 1% v/v acetic acid solution at room temperature and agitated for 24 h. Then the pH of chitosan solution was adjusted to 4.5 by using 5 N NaOH, and a 0.45 μm syringe filter was employed for filtration. *P. harmala* leaves extract (10 mg) was dissolved in CS solution by dissolving the plant leaves extract in a minimum amount of absolute ethanol before it was dissolved in 5 ml of CS solution. 0.5 mg/ml of TPP was dissolved in deionized distilled water to aid in the cross-linking ionic gelation process. 5 ml of CS solution containing plant leaves extract (2 mg/ml) were mixed with 5 ml of TPP solution (0.5 mg/ml) drop-wise to produce *P. harmala* leaves extract dispersions (*P. harmala*-Dis_a_) with a final concentration equivalent to 1 mg/ml plant leaves extract. The solutions were left overnight after being stirred for 30 min at room temperature and 1200 rpm. 0.1 ml of Tween 80 (1%) was added after 24 h to prevent the particles from clumping together. The synthesis process is shown as diagram in Fig. S1a.

#### Preparation of *P. harmala* leaves extract-loaded zinc oxide nanoparticles (*P. harmala*-ZnO NPs)

For synthesis of (ZnO) nanoparticles, a modified simple co-precipitation method was employed^24^. Firstly, blank ZnO NPs were synthesized by preparation of 0.5 M aqueous solution of zinc acetate dehydrates at ambient temperature. Then, 0.5 M solution of sodium hydroxide was added to the zinc acetate dehydrate solution (pH adjusted to 7) with constant stirring (magnetic stirrer) for 2 h at (60 °C) for reduction into Zn^2+^ ions, to achieve light milky suspension of ZnO NPs. White precipitate appeared which indicated synthesis on blank ZnO NPs. Lastly, the aforementioned suspension was repeatedly cleaned with ethanol and deionized water to get rid of extra zinc acetate and other contaminants. It was then dried in a vacuum oven set at 60 °C for 24 h (Fig. S1b).

To create *P. harmala*-ZnO NPs, (10 mg) *P. harmala* leaves extract was added into (10 mL) of aqueous suspension of blank ZnO Nps in a ratio 1:10 was mixed at constant stirring for 1 h at (40 °C temp)^48^. The final concentration of plant extract in *P. harmala*-ZnO-Dis_b_ was (1 mg/ml)^48^, followed by 10 min sonication at ambient temperature. The resultant dispersions were refrigerated at 4 °C, sealed, and shielded from light (Fig. S1c).

#### Preparation of *P. harmala* leaves extract-loaded Chitosan–zinc oxide nanoparticles (*P. harmala*-CS-ZnO NPs)

*P. harmala-CS-ZnO NPs* were made using the previously described simple co-precipitation technique at ambient temperature, and then chitosan was added for coating^24,48^. In brief, the dried blank ZnO Nps was hydrated and mixed with 10 mg (*P. harmala*) leaves extract for one hour at 40 °C using only 5 mL of deionized distilled water and the final concentration of plant extract in the formed *(P. harmala*-ZnO-Dis) was (2 mg/ml)^48^, followed by 10 min sonication at ambient temperature. Then the obtained (5 ml) dispersions of (*P. harmala*-ZnO-Dis) were placed drop-wise with stirring in a beaker containing five milliliter (2 mg/ml) chitosan solution (10 mg chitosan dissolved in 1% acetic acid and stirred for 24 h) to create final *P. harmala*-CS-ZnO NPs dispersions (*P. harmala*-CS-ZnO-Dis_c_) with a final concentration of plant extract equal (1 mg/ml). The solutions were stirred at room temperature and at 1200 rpm for 30 min before being left overnight. These dispersions were then sealed, kept out of the light, and kept in a refrigerator at 4 °C (Fig. S1d).

#### Preparation of *P. harmala* leaves extract-loaded TPP-Chitosan-zinc oxide nanoparticles (*P. harmala*-TPP-CS-ZnO NPs)

*P. harmala*-CS-ZnO NPs was produced according to the previous methods^24,48^ and further coated by (TPP) for the preparation of *P. harmala*-TPP-CS-ZnO NPs. Briefly, 5 ml of (*P. harmala*-ZnO-Dis_b_) was placed drop-wise with stirring in a beaker containing 2.5 milliliter (4 mg/ml) chitosan solution. After 24 h stirring, 0.1 ml of Tween 80 (1%) was added to avoid aggregation. A 2.5 ml of TPP solution (1 mg/ml) were dropped into 7.5 ml of (chitosan solution with *P. harmala*-ZnO plant extract) to promote crosslining and finally create *P. harmala*-TPP-CS-ZnO dispersions (*P. harmala*-TPP-CS-ZnO -Dis_d_) with a final concentration equal (1 mg/ml) plant extract. Following 30 min of stirring at room temperature and 1200 rpm, the solutions were left overnight Fig. S1e.

The final composition and all identification for the previous four nano-formulations are summarized in Table 1 and the schematic presentations of all previous nano-formulations were illustrated in Fig. S1.

**Table 1:** The composition of *P. harmala* leaves extract loaded Nano-dispersion formulations.

Formulation code	CS^a^ (mg/ml)	TPP^a^ (mg/ml)	Tween 80 (%v/v)	*P*. harmala^b^ (mg/ml)	ZnO^b^ (mg/ml)
Blank ZnO NPs	–	–	–	–	10
*P. harmala*- CS NPs	1	0.25	0.1	1	–
*P. harmala*- ZnO NPs	–	–	–	1	10
*P. harmala*-CS-ZnO NPs	1	–	–	1	10
*P. harmala*-TPP-CS-ZnO NPs	1	0.25	0.1	1	10

All formulations contain *P. harmala leaves extract* (1 mg/ml) with final 10 ml volume. ^a^(CS solution : TPP solution = 1:1). CS, chitosan; TPP, tripolyphosphate. ^b^The concentration in 10 ml volume with a ratio (*P. harmala*: ZnO = 1:10) ZnO, Zinc Oxide nanoparticles.

### Characterization of the nano-formulations

#### Colloidal characterization of *P. harmala* -loaded (CS NPs, ZnO NPs, CS-ZnO NPs, TPP-CS-ZnO NPs) as dispersion

The shape, size distribution, zeta potential, particle size, entrapment efficacy (%EE), and loading capacity (% LC) of four *P. harmala* leaves extract nano-formulations and the blank formulas (ZnO NPs) were determined.

Six formulations were analysed for zeta-potential (ZS), dispersion by polydispersity index (PDI), and particle size (PS) using Zetasizer (Malvern, UK). The means and standard deviations (SD) were calculated after each participant completed three measurements.

#### Entrapment efficiency (EE) and loading capacity (LC) of *P. harmala* leaves extract

When measuring free (un-entrapped) *P. harmala* that was isolated from these four colloidal dispersions (CS NPs, ZnO NPs, CS-ZnO NPs, TPP-CS-ZnO NPs), the percentage EE and LC were determined by separation of the formed NPs from the medium by using centrifugation at 14,000 rpm for 30 min at 4 °C^49^. Four colloidal dispersions’ NPs had settled, and their supernatant contained free drug. Utilizing the UV-Shimadzu-spectrophotometer (Japan), the supernatant for *P. harmala* leaves extract at λ_max_ = 324 nm was analyzed. The experiment was carried out three times with the specified mean values. The NPs’ percentage (EE%) and (LC%) were calculated using the following equations:

$$ \frac{ \text{EE \%=Total} \ P.harmala\text{leaves \ extract \ added}{ \ - \ }\text{Free} \ P.harmala \ \rm{leaves \ extract}}{\text{Total} \ P.harmala\rm{leaves \ extract \ added}}\times 100$$

$$ \frac{ \text{LC \%=Total}  P.harmala\text{leaves \ extract \ added}{ \ - \ }\text{Free} \ P.harmala \rm{leaves \ extract}}{\text{Nanoparticles weight} \ }\times 100$$

Where the total *P. harmala* leaves extract represents the whole drug concentration added to the system, and the free *P. harmala* leaves extract represents the concentration of free drug (within the supernatant).

#### Fourier transform-infrared spectrometry (FT-IR)

Fourier transform-infrared (FT-IR) spectra for pure raw *P. harmala* leaves extract, pure chitosan, pure TPP, freeze-dried blank ZnO and freeze dried *P.harmala* nanoparticles were detected using FTIR Spectrometer (Agilent Cary 630, Malaysia) to analyze the chemical structure and functional groups of the prepared nanoparticles. The FT-IR spectra were obtained in the spectral region of 400–4000 cm^−1^.

#### Morphology

Using transmission electron microscopy (TEM), samples of herbal extract entrapment within the four different NPs formulations were examined and photos were taken. The size and internal morphology of the generated *P. harmala* CS NPs, Blank ZnO NPs, *P. harmala*-ZnO NPs, *P. harmala* CS- ZnO NPs, *P. harmala* TPP-CS- ZnO NPs were examined using (TEM, JEOL JEM1400-PLUS, Joel Ltd., Tokyo- Japan).

#### In vitro release study

The in vitro release investigation of the four *P. harmala* leaves extract nano-formulations was evaluated using the dialysis-bag diffusion method in contrast to *P. harmala* leaves extract solution (Sol). The release rates were tested using a dissolution media containing phosphate buffer saline (PBS) at pH 6.8. for *P. harmala* solution, *P. harmala*-CS NP susp, *P. harmala*-ZnO NP susp, *P. harmala*-CS-ZnO NP susp and *P. harmala*-TPP-CS-ZnO NP susp^50^. One ml of test *P. harmala* herbal extract formula contained which contains 1 mg was put inside a dialysis bag that had been previously wet and sealed on both ends.

According to an assessment, the *P. harmala* leaves extract’s solubility in the release medium was 12.7 mg/ml, enabling the sink-condition to be maintained. This is why the dialysis bag was only submerged in five millilitres of dissolving solution (PBS at pH 6.8) within the glass beaker (receptor compartment). The temperature was kept at 37 ± 0.5 °C while being shaken at 100 rpm using a thermostat-controlled water bath with a horizontal shaker^50^. At predetermined intervals (1, 2, 4, 6, 12, and 24 h), all 5 ml of the receiving media were removed and replaced with an additional 5 ml of fresh medium in order to maintain sink-condition. The *P. harmala* leaves extract drug content of the samples was determined using spectrophotometry at a wavelength of 324 nm (λ_max_). The data were analysed using linear regression equations based on the calibration curves of *P. harmala leaves extract* in a dissolution media (with concentration ranges of 10–100 µg/ml). Three measurements were made for each measurement.

### In vitro antiviral evaluation of the effect of different *P. harmala* leaves extract nano-formulations on herpes simplex 1 (HSV-1)

#### MTT cytotoxicity assay for *P. harmala* leaves extract and its nano-formulations

First, MTT cytotoxicity assay for *P. harmala* leaves extract and its nano-formulations were conducted to clarify safe and effective dosages of examined substances for the anti-viral activity assessment and prevent confusion between cytotoxicity and potential bioactivity.

The detailed description of MTT cytotoxicity assay^51^ was provided in the supplementary material.

#### Inhibitory concentration 50 (IC_50_) determinations for *P. harmala* leaves extract and its nano-formulations

In 96-well tissue culture plates, 2.4 × 10^4^ Vero-E6 cells were distributed in each well and incubated overnight at a humidified 37 °C incubator under 5% CO_2_ conditions^52^. The cell monolayers were then washed once with 1× PBS and subjected to virus adsorption [hCoV-19/Egypt/NRC-03/2020 (Accession Number on GSAID: EPI_ISL_430820)] for 1 h at room temperature (RT). The cell monolayers were further overlaid with 100 µl of DMEM containing varying concentrations of the test compounds. Following incubation at 37 °C in a 5% CO_2_ incubator for 72 h, the cells were fixed with 100 µl of 4% paraformaldehyde for 20 min and stained with 0.1% crystal violet in distilled water for 15 min at RT. The crystal violet dye was then dissolved using 100 µl of absolute methanol per well, and the optical density of the color was measured at 570 nm using an Anthos Zenyth 200 at RT plate reader (Anthos Labtec Instruments, Heerhugowaard, Netherlands). The IC_50_ of the compound is the concentration required to reduce the virus-induced cytopathic effect (CPE) by 50%, relative to the virus control^52^.

#### Plaque reduction assay for *P. harmala* leaves extract and its nano-formulations

VERO-E6 cells at a density of 1 × 10^5^ cells per well in 6-well plates. A confluent monolayer should form after an overnight incubation at 37 °C in a humidified environment with 5% CO_2_. Apply 0.1 PFU/cell of HSV-1 multiplicity of infection (MOI) to the cell monolayers. To enable viral adsorption, incubate for one hour at 37 °C. After removing the inoculum, give the cells two PBS washes. The six test formulations should be added in serial dilutions at the specified concentrations. Add a negative control (uninfected cells) and a positive control (virus-infected cells not receiving treatment). Prepare a MEM mixture with 0.8% agarose and 2% fetal bovine serum (FBS). Cover each well with the agarose mixture to preserve the cells while permitting the creation of plaque. To enable plaque development, incubate the plates at 37 °C for 48 h. For fifteen minutes, fix the cells with 10% formaldehyde. After giving the plates a PBS wash, stain them for fifteen minutes using crystal violet. After removing any excess stain, let the plates air dry. Using a dissecting microscope, determine how many plaques are present in each well^53^.

Calculate the percentage of viral inhibition using the formula^53^:

$${\text{\% Inhibition = (1}} - {\text{(average of tested sample - treated cells) /(average of control cells)}} \times {\text{100}}$$

The sample tested means blank nano-formulations or *P. harmala leaves extract* whatever it is in raw form or in nano-formulation.

### Mode of action against herpes simplex virus type 1 (HSV-1)

The possible mode of action of *P. harmala* nano-formula with the most leading antiviral observation according to Plaque Reduction Assay against HSV-1 strain and examined at three different stages of the virus propagation cycle and based on three main possible modes of action: (i) Inhibition of budding and viral replication^54^, (ii) The ability of sample to inhibit the attachment of the virus to infected cells-membrane fusion which known as blocking the viral entry (viral adsorption)^55^, and (iii) The direct effect of the sample to inactivate the virus viability (virucidal activity)^56^. Additionally, the above-mentioned mode of action could account for the recorded antiviral activities either independently, or in combinations.

### Investigated correlation

Investigating the relationship between in vitro pharmaceutical and in vitro antimicrobial (antiviral) processes involved plotting the mean percent D.E. (dissolution efficiency), difference factor (*f1*) or similarity factor (*f2*) against percent inhibition of Herpes simplex type-1 (HSV-1). A linear regression analysis was conducted to estimate the robustness of the correlation and evaluate its strength and significance. The *p-value* and R^2^ were calculated^43,57–59^.

### Statistical analysis

GraphPad Prism software (version 7.0) was used to analyze the data. Each in vitro test was run three times, and the results were given as the mean ± SD. ANOVA was employed to statistically assess the data. The threshold for statistical significance was set at a difference with a p-value of ≤ 0.05. The Student–Newman–Keuls multiple comparison test was performed after a one-way ANOVA to compare the various experimental groups. The *P-value* of less than or equal 0.05 was used to determine the significance level of the results.

## Results and discussion

### Phytochemical investigation

#### Chemical composition of *P. harmala* leaves extract through UPLC-MS/MS analysis

The chemical profile of *P. harmala* leaves extract was illuminated using UPLC-MS/MS analysis where a total of 25 prominent peaks spanning varying chemical class primarily alkaloids (*β*-carbolines and quinazolines), phenolic acid, flavonoids, fatty acids and terpenoids were recorded and characterized based on some mass measurements as retention time, quasi-molecular ions along with informative diagnostic mass fragments in comparison with reference standards and relating literature data. Table 2 recapitulated the full list of characterized compounds prevalently exiting in *P. harmala* leaves extract besides their detailed structural data regarding retention time, quasi-molecular ions, the distinguishing fragments, molecular formula as well as chemical classes. Also, base peak chromatograms (BPCs) of *P. harmala* leaves extract in both positive and negative ion modes are divulged in Fig. S2.

**Table 2:** List of characterized compounds in *P. harmala* leaves extract through UPLC-MS/MS analysis.

No.	Rt (min.)	Identified compounds	Precursor ions	Chemical formulas	Main fragments (Da)	Chemical classes	References
1	0.76	Gamma amino butyric acid (GABA)	104.2 M + H	C_4_H_9_NO_2_	87-76	Amino acids	^35^
2	1.28	Citric acid	191.2 M-H	C₆H₈O₇	163-135-117	Organic acids	^36^
3	1.7	Protocatechuic acid	153.3 M-H	C_7_H_6_O_4_	109	Phenolic acids	^60^
4	2.3	Hydroxy benzoic acid	137.3 M-H	C_7_H_6_O_3_	93	Phenolic acids	^60^
5	3.3	Coumaric acid	163.3 M-H	C_9_H_8_O_3_	147-119	Phenolic acids	^61^
6	4.2	Peganone1	283.3 M-H	C_16_H_12_O_5_	265-250-237-209	anthraquinones	^62^
7	5.5	Pegamine	203.3 M-H	C_11_H_12_N_2_O_2_	187-120	Alkaloids (quinazolines)	^63^
8	5.8	Harmine	211.2 M-H	C_13_H_12_N_2_O	198-170	Alkaloids (β-carboline)	^63^
9	6.3	Vasicine (peganine)	189.3 M + H	C_11_H_12_N_2_O	171-154	Alkaloids (quinazolines)	^64^
10	6.7	Peganone2	267.2 M-H	C_16_H_12_O_4_	249-234-221-193	anthraquinones	^62^
11	5.5	Pegamine dimer	409.3 M + H	C_22_H_24_N_4_O_4_	387-205-189	Alkaloids (quinazolines)	^63^
12	6.2	Acacetin-O-rutinoside (Linarin)	593.3 M + H	C_28_H_32_O_14_	447-285-272-244	Flavonoids	^65^
13	7.4	Acacetin	283.3 M-H	C_16_H_12_O_5_	270-242-153-133	Flavonoids	^66^
14	9.7	Harman	183.3 M + H	C_12_H_10_N_2_	168	Alkaloids (β-carboline)	^64^
15	9.9	Harmol	199.2 M + H	C_12_H_10_N_2_O	171-131-73	Alkaloids (β-carboline)	^64^
16	10.8	Harmalidine	255.3 M + H	C_16_H_18_N_2_O	225-	Alkaloids (β-carboline)	^63^
17	13.4	Rosmanol	347.2 M-H	C_20_H_26_O_5_	301-231	Terpenoids	^67^
18	14.8	Palmitic acid	255.2 M-H	C₁₆H₃₂O₂	211-74	Fatty acids	^68^
19	15.4	Stearidonic acid	277.3 M + H	C_18_H_18_O_5_	255-233-162	Fatty acids	^37^
20	16.7	Hydroxy linolenic acid	295.3 M + H	C_18_H_30_O_3_	279-235-59	Fatty acids	^69^
21	17.4	δ-Tocotrienol	395.3 M-H	C_27_H_40_O_2_	380-135	Diterpenoid “tocopherol”	^37^
22	18.8	Hydroxy linoleic acid	295.3 M-H	C_18_H_32_O_3_	279-235-61	Fatty acids	^70^
23	19.2	Linolenic acid	279.3 M + H	C_18_H_30_O_2_	235-59	Fatty acids	^16^
24	20.5	β-Tocopherol	417.3 M + H	C_28_H_48_O_2_	402-266-151-123	Diterpenoid “tocopherols”	^71^
25	24.3	β-Sitosterol	413.2 M-H	C_29_H_50_O	395-273-161	Phytosterols	^16^

### Network pharmacology analysis

#### HSV-1 target genes of *P. harmala* leaves extract compounds

With the intention to catalogue the complex picture of drug pharmacological actions aligning with the aspects of multi-compound, multi-target and multi-pathway effects of medicinal plants in manging diseases, a new-cutting edge approach “network pharmacology” has been successfully implemented^16^.

First of all, the annotated compounds retrieved from UPLC-MS analysis were assessed by the ADME parameters of OB and Lipinski’s Rule (LR) to pre-screen and pick out the compounds with favorable pharmacokinetic features where the compounds that fulfilled the screening criteria of OB ≥ 30% and at least three of Lipinski’s Rule (LR) were selected for network pharmacology analysis as depicted in Table S1.

**Fig. 1:**
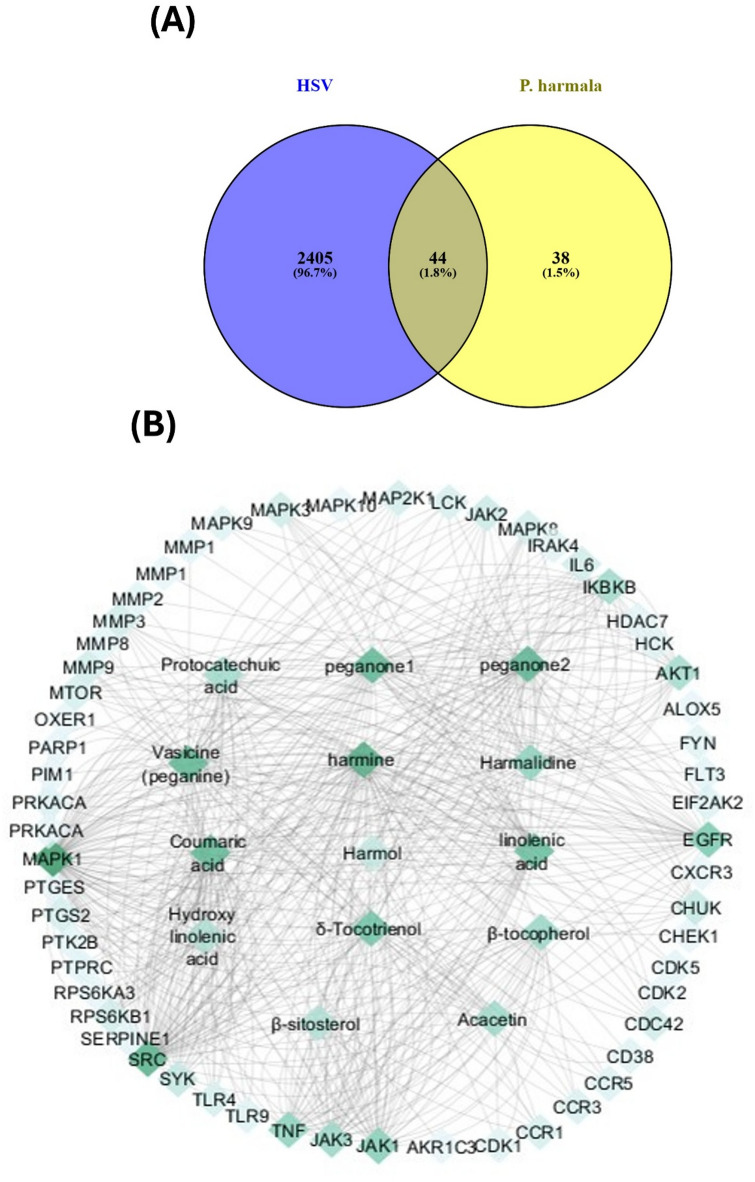
(**A**) Venn diagram illustrating the number of common genes shared between *P. harmala*-related genes and Herpes simplex virus (HSV-1) disease genes. (**B**) *P. harmala*-components-targets interactions network where the degree value of the nodes is shown on a color-coded scale from deep to light green.

In the current research, the genomic databases including Therapeutic Target Database (TTD) and GeneCards were utilized to profile all genes associated with HSV infection where a total of 2449 genes were pinpointed. In the meantime, 25 assigned compounds from *P. harmala* leaves extract were submitted to STITCH 5.0 and Swiss Target Prediction databases for target genes prediction. After discarding non-relating genes and compounds using a Venn mapping tool, a total 44 targets of 14 compounds were intersected with HSV-1 infection (Fig. 1A). Crucially, the molecular interactions between candidate compounds and their target genes are calculated and expressed by “combined score” where high scores indicate stronger interactions. Genes with a combined score greater than 0.7 were thus chosen as the primary targets and a compound-gene network was created using Cytoscape 3.7.2. revealing the functional interactions between the candidate compounds and their related targets on HSV mitigation.

As presented in the compound-gene network (Fig. 1B), there were 58 nodes and 513 edges, with 14 components and 44 potential targets associated with HSV-1. On average, there were 3.717 connected targets directly acting on each compound, suggesting multiple drug-target interactions. Notably, as Table S2 summarized, the Uniprot database was utilized to obtain referenced information about the gene names of “*Homo sapiens*” species and their biological functions. Based on degree analysis, MAPK 1, SRC, EGFR, JAK1, IKBKB and TNF were found to be the highly correlated putative genes with the efficacy mechanism of *P. harmala* compounds on HSV-1 mitigation as depicted in Fig. 1B; Table 3 which steadily dissected the topological parameters of the potential targets. While the respective forecasted compounds directly acting on these hub targets and might be the potentially active compounds synergistically work against HSV-1 infection included harmine, peganone 2, peganone 1, vasicine, coumaric acid, and linolenic acid.

Under the guidance of relevant literature, β-carboline alkaloids primarily harmine have been credited with exhibiting a promising viricidal potency against the influenza A and H5N1 viruses via interfering with viral replication^72^. It exhibits antiviral activity by targeting host cellular pathways, rather than directly attacking the virus itself. It inhibits viral replication by interfering with RNA processing, specifically by targeting SR kinases^73^. Additionally, harmine can modulate the NF-κB and MAPK pathways, which are crucial for viral replication and the host’s inflammatory response^72^. It can also reduce oxidative stress induced by viral infections and inhibit the expression of viral genes. Equally important, *P. harmala* anthraquinones primarily peganone 1, and peganone 2 have been reported to inhibit Human cytomegalovirus (CMV), a genus of viruses in the order Herpes virales, via damaging cell membranes, potentially interfering with viral entry and, inhibiting the viral replication through blocking the viral DNA synthesis^74,75^. Relatedly, a leading work has evidenced the noteworthy antiviral activity of vasicine (peganine) against COVID-19^76^. Beside this, a growing body of research has shown that phenolic acids primarily gallic, coumaric and caffeic acids have been reported to effectively impedes HSV-1 infection via repressing viral adsorption and entry, while also curbing the expression of antiviral cytokines^77^. They also exhibit antiviral activity by direct acting on the NF-κB and MAPK pathways, thereby reducing the production of inflammatory cytokines and potentially hindering viral replication^77^. Further, a recent leading study has demonstrated the remarkable antiviral potency of linolenic acid against Zika virus (ZIKV), influenza virus, HSV-1 and coronavirus infectivity dose-dependently via disrupting the membrane integrity of the virions^78^.

**Table 3:** *P. harmala* leaves extract main active ingredient’s targets network node topological parameters.

Gene name	Betweenness centrality	Closeness centrality	Degree	Neighborhood connectivity	Topological coefficient
MAPK1	0.149678	0.375	90	8.833333	0.211712
SRC	0.211616	0.418605	62	9.857143	0.201299
EGFR	0.173478	0.391304	35	9.428571	0.221805
JAK1	0.054471	0.336449	31	9.25	0.317308
TNF	0.054649	0.305085	29	6.666667	0.354167
JAK3	0.063053	0.330275	25	7.2	0.248
IKBKB	0.003703	0.290323	23	8.5	0.576923
AKT1	0.003565	0.295082	22	12.5	0.638889
SYK	0.008344	0.305085	12	12.5	0.547619
CHUK	0	0.27907	11	13	0
JAK2	0.025721	0.305085	10	8.333333	0.407407
MAPK3	0.037632	0.327273	9	11	0.416667
IL6	0	0.255319	8	8	0
MAPK8	0.027367	0.288	8	6.666667	0.404762
CDC42	0	0.27907	8	13	0
RPS6KB1	0.003565	0.295082	8	12.5	0.638889
MMP3	0.042035	0.333333	8	10	0.333333
MMP9	0.024357	0.324324	8	9.5	0.354167
MAP2K1	0	0.28125	7	11	0
CCR1	0.005168	0.24	6	4.5	0.583333
MTOR	0	0.274809	6	11	0
PTGS2	0	0.276923	6	11	0
TLR4	0.006047	0.288	6	9	0.571429
LCK	0.015327	0.307692	6	10.66667	0.439394
CCR3	0.004713	0.26087	5	6.5	0.611111
CCR5	0	0.236842	4	8	0
PRKACA	0	0.27907	4	13	0
EIF2AK2	0	0.270677	4	14	0
CDK1	0.005166	0.268657	3	6	0.555556
MAPK9	0	0.274809	3	11	0
IRAK4	0	0.27907	3	13	0
CD38	0.008326	0.3	3	10	0.529412
HCK	0	0.270677	3	14	0
FYN	0	0.276923	3	11	0
TLR9	0	0.276923	3	11	0
MMP8	0	0.236842	2	8	0
FLT3	0	0.25	2	6	0
MMP2	0.002639	0.285714	2	9	0.571429
CDK5	0	0.27907	2	13	0
PTPRC	0	0.27907	2	13	0
HDAC7	0	0.270677	2	14	0
MMP1	0.004707	0.305085	2	12.5	0.605263
PTK2B	0	0.276923	2	11	0
SERPINE1	0	0.248276	2	7	0
CXCR3	0	0.196721	1	4	0
CHEK1	0	0.236842	1	8	0
MAPK10	0	0.230769	1	5	0
RPS6KA3	0	0.229299	1	5	0
PTGES	0	0.255319	1	8	0
OXER1	0	0.255319	1	8	0
MMP1	0	0.25	1	6	0
PIM1	0	0.28125	1	11	0
PRKACA	0	0.27907	1	13	0
AKR1C3	0	0.276923	1	11	0
ALOX5	0	0.276923	1	11	0
CDK2	0	0.248276	1	7	0
PARP1	0	0.248276	1	7	0

**Fig. 2:**
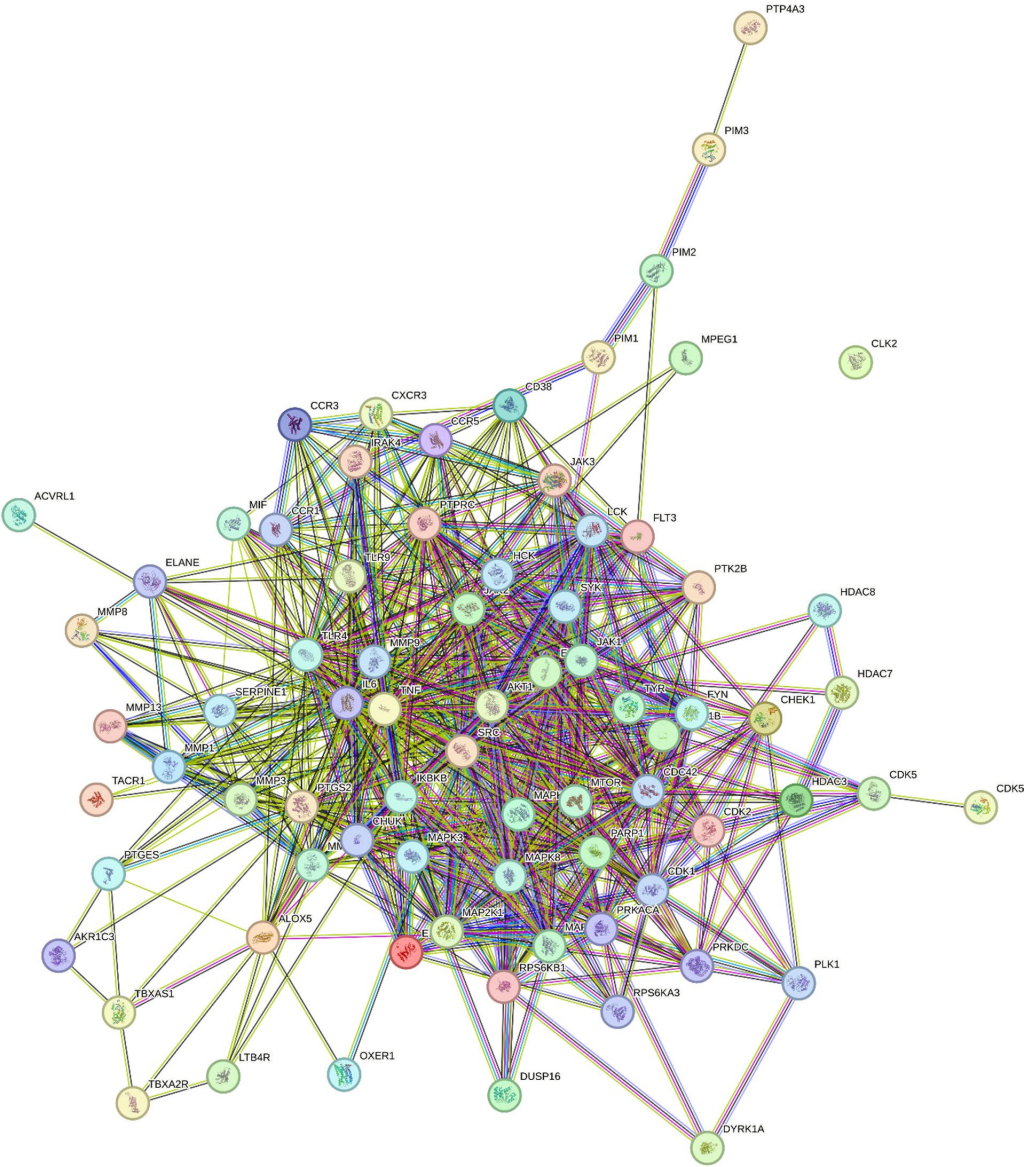
Protein–protein interaction (PPI) diagram of target genes related to HSV-1 disease and *P. harmala* leaves extract compounds.

In the same regard, the HSV main target genes were submitted to STRING ver. 10.5 database for protein-protein interactions (PPIs) network establishment gaining a comprehensive view for the molecular interactions among these proteins. As depicted in Fig. 2, PPI network comprising high-confidence proteins interactions with combined score > 0.7 revealed 44 nodes reflecting biologically significant genes and 542 edges of protein-protein interactions where MAPK 1, SRC, EGFR, JAK1, IKBKB, AKT1, TNF, MMP9, and IL6 were centrally placed in PPI map affirming their multifaceted roles in HSV pathogenesis.

In accordance with compelling evidence, Mitogen-activated protein kinases (MAPKs), in particular MAPK 1 play an integrative role in the cellular immune response to virus infections which is typically manifested by regulating virus cytokines production and inflammation^79^. For the sake of clarity, DNA and RNA viruses interfere with host MAPK signalling pathways in order to create a permissive environment for their productive replication, cell division and latency by incapacitating host antiviral defense^79^. Relatedly, tyrosine-protein kinase (SRC) has been well-reported as exhibiting a crucial role in optimizing antiviral immune responses^80^. When dysregulated, SRC participates in augmenting viral yields over the course of viral replication of hepatitis B and HSV^80^. Importantly, accumulating evidence has underscored the fundamental role of epidermal growth factor receptor (EGFR) in the entry, replication and effective propagation of multiple viral species as well as the subversion of innate responses^81^. In brief, EGFR, which is anchored on the plasma membrane, is essential for coordinating viral entry process, replication and immunological response evasion^81^. Further, EGFR trafficking is essential for virus-host interactions allowing successful propagation of various viral agents into a variety of intracellular organelles primarily lysosomes, mitochondria, and the nucleus of the host cells^81^. So far, understanding EGFR involvement in diverse cellular pathways and viral pathogenesis may help explore innovative antiviral remedies. Essentially, Janus kinase (JAK 1) serves as a main actor of local and systemic inflammatory responses post viral infections which in turn block viral replication via the production of downstream antiviral IFN-stimulated genes (ISGs) and proinflammatory mediators^82^. When hyper-activated, it excessively stimulates innate immunity resulting in overproduction of IFNs, proinflammatory cytokines concluding with cytokine storms typically implicated in multiple-organ damage and death^82^. The currently best-reported function of serine/threonine kinase (AKT) is to underlie different cellular responses as cell growth, protein synthesis and inflammation to rectify viral infections. Worth noting, Herpesviruses produce numerous proteins that interact with the PI3K/Akt pathway to ease viral replication, latency and reactivation^83^. Undoubtedly, Tumor necrosis factor and Interleukin 6 (TNF and IL-6), transiently generated as downstream proteins post viral infections to regulate host immune reactions^84^. However, dysregulated expression of these cytokines pathologically leads to multiple organ dysfunction onset^84^.

Thereby, understanding the intricate interplay between viruses and these putative targets is supreme for devising effectual treatment strategies to mitigate HSV infections.

#### Forecasting the main signalling pathways of the top-ranked genes of *P. harmala* compounds

To fully elucidate the multi-waved molecular basis underlying the anti-HSV potential of *P. harmala* compounds, KEGG pathway analysis was established mapping the interactions between the target genes and HSV signalling pathways.

**Fig. 3:**
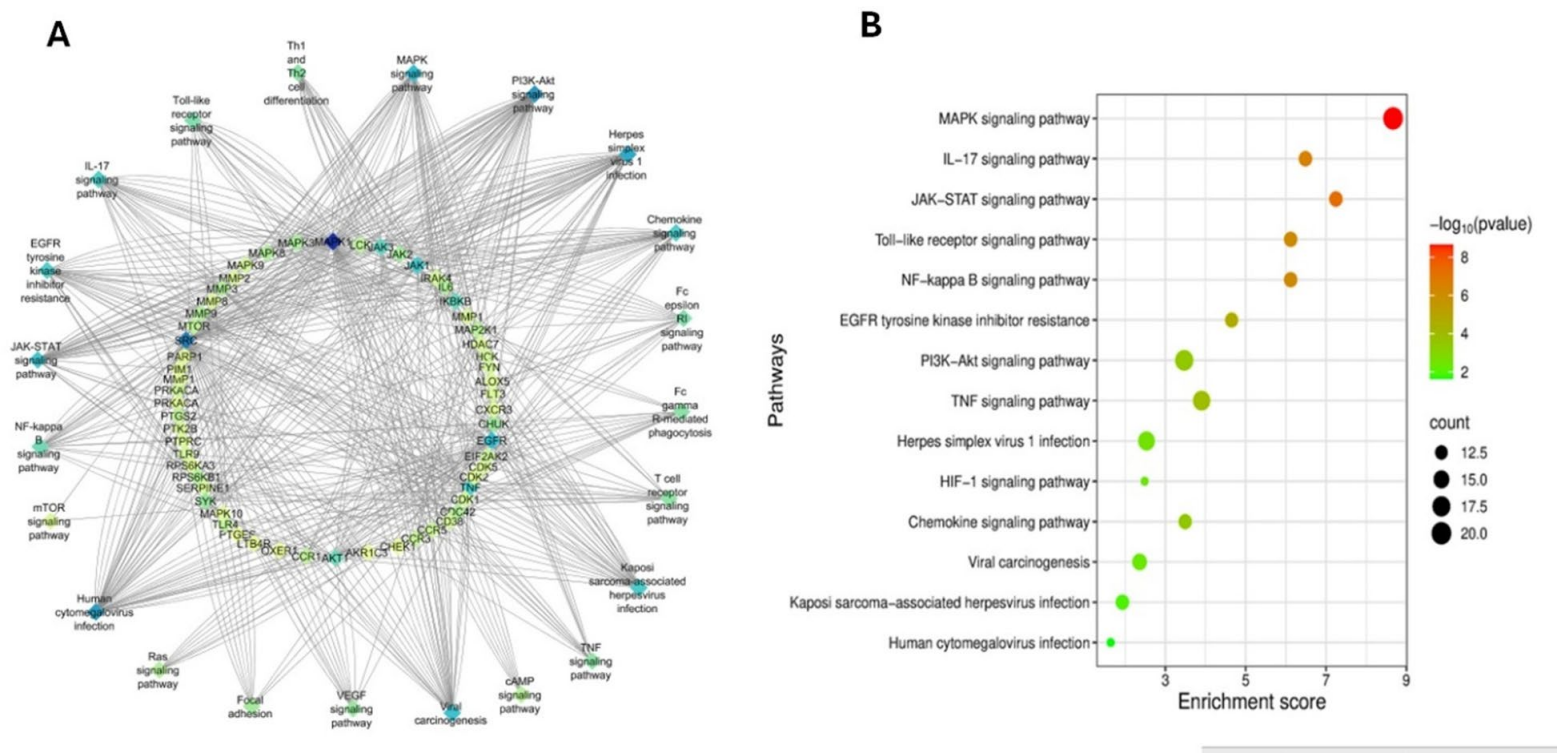
(**A**) KEGG pathways - targets network where the color of the node reflects the degree value; the more deeply the color shifts from blue to yellow, the more significant the genes and pathways. (**B**) The top 15 KEGG signaling pathways based on enrichment score^39,40^.

Figure 3A and Table S3 dissected the highly relevant KEGG pathways to HSV infections with false discovery rate < 0.05. Among this, MAPK signalling pathway, PI3K-Akt signalling pathway, chemokine signalling pathway, IL-17 signalling pathway, JAK-STAT signalling pathway and EGFR tyrosine kinase inhibitor resistance were ranked the highest in their relevance with HSV and co-regulated by the top-listed genes alluded above as clarified in the bubble plot (Fig. 3B).

As stated above, all herpes viruses share the characteristic of being able to cause a persistent infection even while the immune system is functioning^1^. To accomplish this, herpesviruses have developed a number of tactics to inhibit or take advantage of host innate immune signalling pathways facilitating their infections.

In a more targeted approach, earlier reports have elucidated that MAPK signalling pathway is one of notably activated host cellular mechanisms in response to HSV infection. In short, the MAPK pathway is initially activated during viral invasion supressing various metabolic pathways pivotal for viral replication^17,83^. In order to further impede viral replication cycle, the MAPK pathway activates its downstream transcription factor, STAT1. However, through host p38-AKT axis commandeering, HSV can circumvent this biphasic antiviral barrier by shielding infected cells from infection-induced apoptosis^80,85^. compelling evidence has consolidated that HSV proteins in the post-entry stage actively induced PI3K-Akt signalling pathway which in turn triggers phosphorylation of different downstream targets facilitating the trafficking of HSV virions among the infected cells^83^. Thus, blocking PI3K-Akt pathway and its downstream agents might be a promising target for treating herpesvirus infections or cancers linked to latent herpesvirus infections. EGFR tyrosine kinase inhibitor resistance pathway plays an essential role in the intricate interactions between viruses and host cells functioning as both enhancer and inhibitor of virus infections, and its action is frequently contingent on the particular virus state^81^. Also, dysregulated EGFR signalling in response to viral invasion is often linked with circumvention of host immunosurveillance, tissue damage and malignancies that conclude with grave complications^81^. In the same context, multiple investigations have demonstrated that IL-17 pathway is tightly implicated during viral infections not only orchestrating the antiviral immune reactions through neutrophil recruitment and activation but also exacerbating virus-induced illnesses^86^. Although the JAK/STAT pathway helps regulate immune responses against viral infections, a vast body of evidence suggests that inappropriate activation or obliteration of this cascade plays a part in viral pathogen replication and pathogenesis^82,87^.

In the light of the above, the blockade of these signalling pathways could potentially serve as rational and targeted treatment strategies against HSV-1 infections.

### Molecular docking analysis

**Fig. 4:**
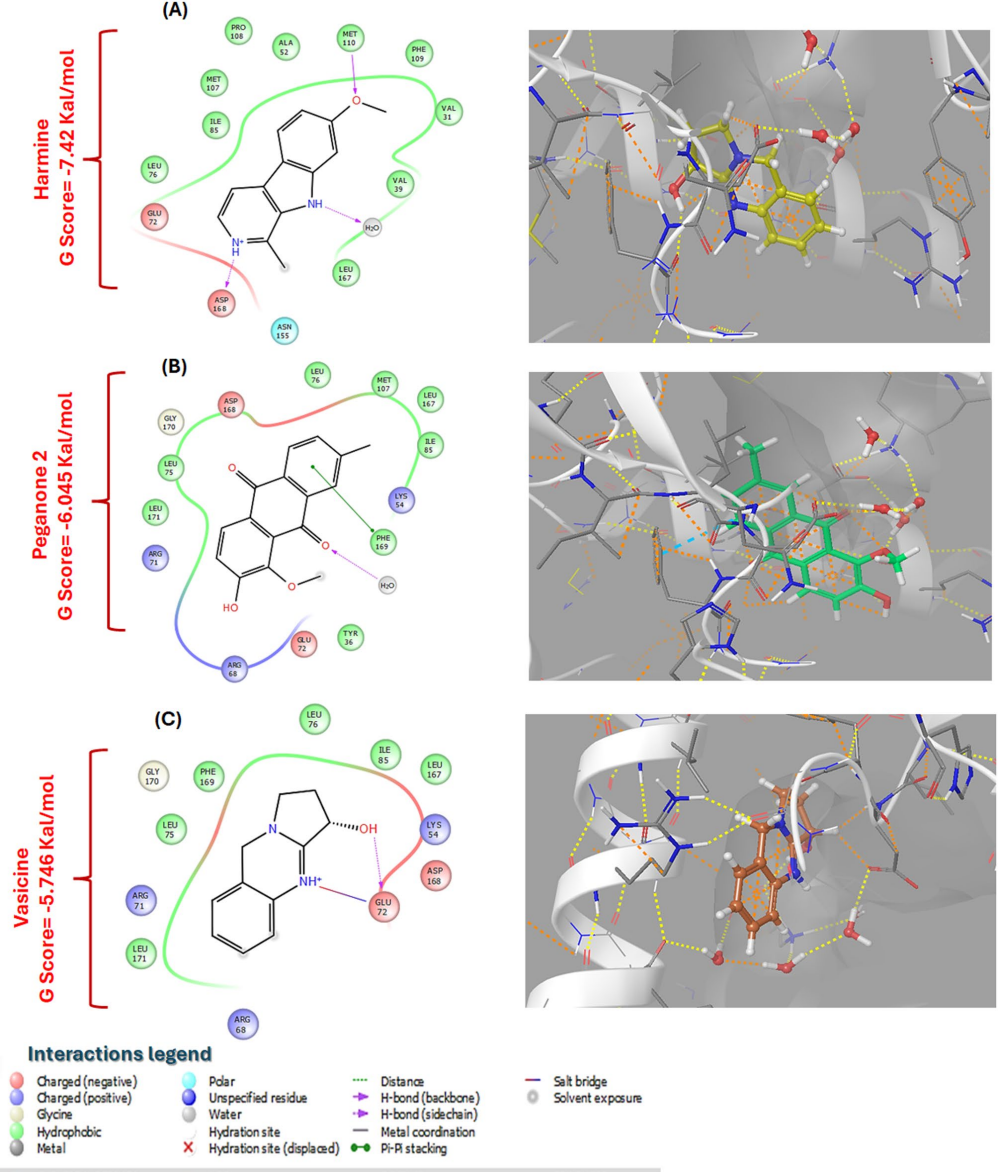
2D and 3D interaction diagrams of harmine (**A**), peganone 2 (**B**), and vasicine (**C**) in the active site of Mitogen-activated protein kinase 1 (MAPK 1) (PBD ID 5EKO).

In the light of network pharmacology findings, molecular docking analysis was conducted to inspect the binding orientations between the top-ranked *Harmala* compounds and MAPK 1 protein. The collected data showed that the candidate compounds exhibited promising binding affinities toward the target protein, with notable binding scores assuring good stability and fitting in the catalytic regions of the protein. In a more specific approach, harmine exerted a significant binding affinity to MAPK 1 protein catalytic site with XP Gscores equal to − 7.74 kcal/mol via forming polar hydrogen bonds and electrostatic interactions with Asp168, Met 110, and Glu 72 as well as hydrophobic interactions with Phe 109, Val 31, Ala 52, Met 107, and Ilu 85 as presented in Fig. 4A. Equally important, peganone 2 was well fitted into MAPK 1 pocket through different binding orientations mainly manifested by electrostatic interactions with Arg 68, Arg 71, Lys 54 and Asp168 beside to van der Waals interactions with Leu 75, Ile 85 and Met 107 (Fig. 4B). Similarly, 2D and 3D diagrams of vasicine (Fig. 4C) displayed its favorable binding tendency toward MAPK 1 catalytic site with a good binding score equal to − 5.746 kcal/mol where the hydroxylic and amino groups were observed to engage in hydrogen and salt bridge interactions with Glu 72 while the residues, including Leu 76, Phe 169, Leu 167, Ile 171, and Leu 75 were noted to be captivated in hydrophobic interactions with the quinazoline core of vasicine possibly orienting vasicine within MAPK 1 binding site and stabilizing the resulting complex Fig. 4C.

### Establishment and evaluation of different nano-formulations loaded with *P. harmala* leaves extract

In the following series of our reasonably constructed workflow, given with network pharmacology findings, which clearly revealed the pharmacological mechanisms of *P. harmala* bioactive compounds in HSV-1 mitigation through pinpointing pathways of interactions between the compounds, main genes, and disease pathways from a network aspect. Even though network pharmacology is a swift and effective strategy for predicting multiple therapeutic targets in multifaceted diseases, our network analysis findings need to be validated experimentally, unlocking correlativity and potential efficacies of *P. harmala* bioactive compounds against HSV-1 infection. Further, with a scope to enhance the efficacy and the therapeutic properties of *P. harmala* bioactive compounds via improving solubility, bioavailability, targeted delivery, and controlled release of active compounds, *P. harmala* leaves extract has been incorporated into nanoparticles subsequently subjected to evaluation scores as depicted in the following subsections. Following that, all nano-formulations established were experimentally evaluated against HSV-1.

### FTIR (Fourier transform Infrared) spectral analysis

FTIR spectroscopy is an auspicious tool in nanotechnology for characterizing the molecular structure and composition of nanomaterials. It is used to identify functional groups, analyze molecular interactions, and assess surface properties of nanoparticles^88^.

In the current study, FTIR analysis was conducted to understand the nanoparticle synthesis, functionalization, and interactions with *Harmala* bioactive compounds. As stated above, *P. harmala* leaves extract is enriched with alkaloids (*β*-carbolines and quinazolines), phenolic acid, flavonoids, fatty acids, and terpenoids which harmoniously interact with the surface of nanomaterials as observed by FTIR spectra inspection.

**Fig. 5:**
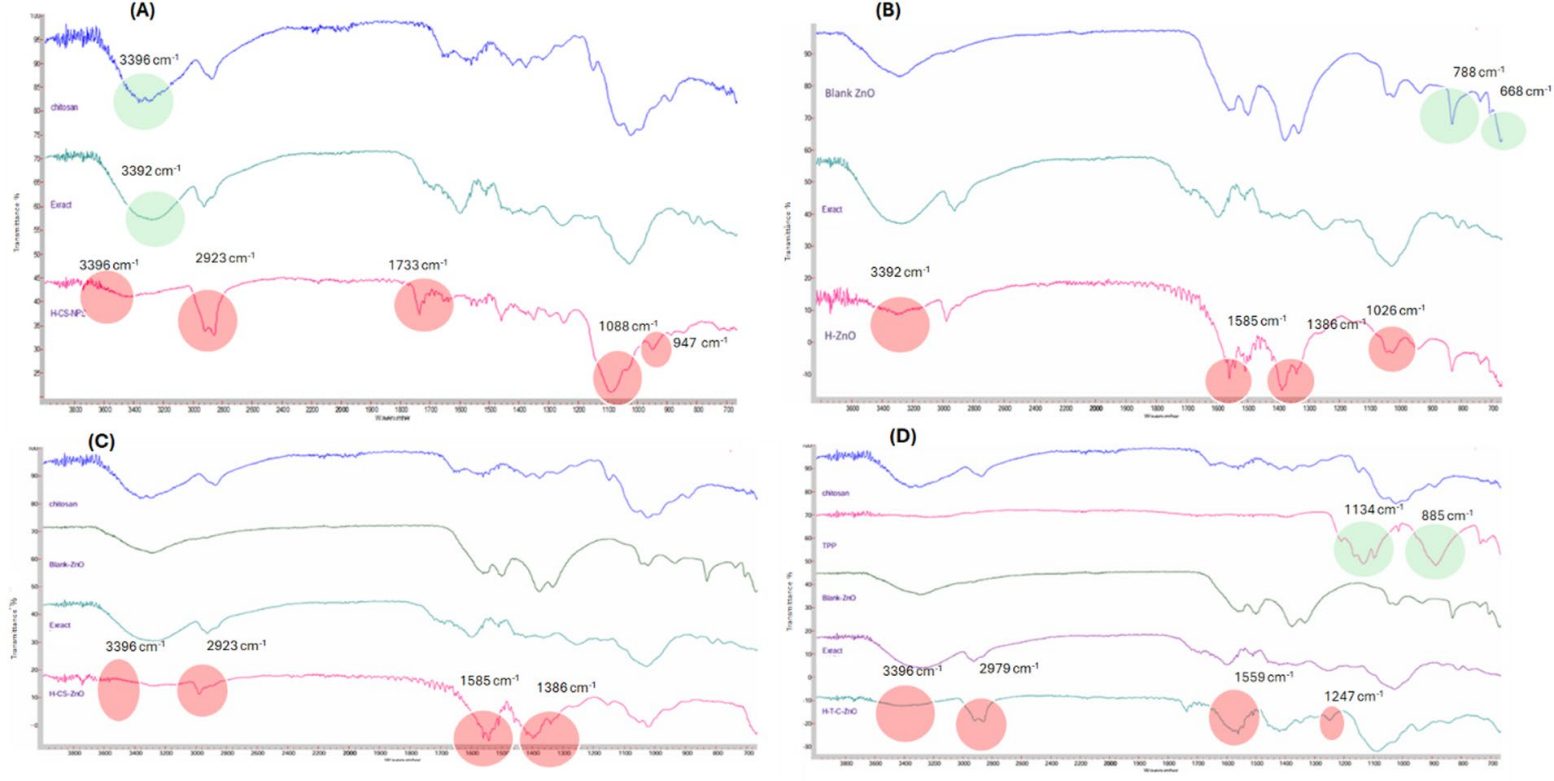
FTIR spectra of different nano-formulations loaded with *P. harmala* leaves extract. (**A**) *P. harmala*-CS-NPs, (**B**) *P. harmala*- ZnO NPs (**C**); *P. harmala* -CS-ZnO NPs, and (**D**) *P. harmala* - TPP-CS-ZnO NPs.

For clarity, the FTIR spectrum of *P. harmala* leaves extract Fig. 5A exhibited distinctive peaks at 3392 cm^− 1^ (O–H), 2923 cm^− 1^ (C–H), 1685 cm^− 1^ (C=O) 1594 cm^− 1^ (N–H) bending, and 1026 cm^− 1^ (C–O) or 812 cm^− 1^ (RCOO) related to alkaloids, flavonoids, and fatty acids, respectively. However, chitosan is characterized with bands at the wavelength of 3396 cm^− 1^, 2872 cm^− 1^, 1647 cm^− 1^, and 1546 cm^− 1^ which are attributable to O–H stretching, symmetric and asymmetric C–H, C=O bond in the *N*-acetyl group, and the amino group bonds, respectively. Further, two sharp peaks at 1058 cm^− 1^ and 891 cm^− 1^ are typically matching with a glycosidic bond and the hydroxyl group. However, the FTIR spectrum of the biosynthesized *P. harmala*- CS NPs witnessed a slight shift with minor change in the intensity of the peak at 2923 cm^− 1^ and appearance of a new band at 1733 cm^− 1^ corresponding to amide linkage between the *Harmala* phenolic compounds and chitosan nanoparticles. In addition, a notable increase in the wavelength number and intensity of the bands at 1026 to 1088 cm^− 1^ and 891 to 947 cm^− 1^, reflecting C–O stretching of amides and successful incorporation of *Harmala* into CSNPs. The broad band at 3390 cm^− 1^ in the CSNPs cannot be observed in *P. harmala*- CS NPs suggesting that the major compounds from the extract were capped or chemically entrapped to the surface of CSNPs Fig. 5A.

The FTIR spectrum of ZnO (zinc oxide) is typically distinguished with a band at 688–800 cm^− 1^ corresponding to the metal-oxygen stretching vibration. Other bands at 2980 and 1430 may be also present due to the presence of surface hydroxyl groups (–OH) and C–N bonds^89^. However, incorporating ZnO into nano-formulations containing *P. harmala* leaves extract revealed a notable spectral shifting from that of the extract and ZnO, indicating the chemical interactions of the extract compounds with the ZnO NPs. More specifically, in the FTIR spectrum of the *P. harmala*- ZnO NPs, the strong absorption bands at 3392 cm^− 1^ (O–H) and 1026 cm^− 1^ (C–O) exhibited a noticeable reduction. Further, the spectrum exhibited a small shift in the vibrational bands of some functional groups like C=O (carbonyl) or N–O (nitro) due to hydrogen bonding interactions with ZnO Fig. 5B. Two prominent bands at 1386 cm^− 1^ and 877 cm^− 1^ were also recorded in the *P. harmala*-ZnO NPs spectrum signifying the successful interactions and efficient stabilization of zinc oxide nanoparticles by different organic moieties existing in the *Harmala* extract.

Comparably, as depicted in the FTIR spectrum of the *P. harmala*-CS-ZnO NPs (Fig. 5C), the two major peaks at 3396 cm^− 1^ and 2923 cm^− 1^ disappeared or exhibited a marked decline, demonstrating that the main biomolecules in the extract were capped or chemically entrapped into the surface of chitosan and ZnO NPs. However, the peaks at 1585 cm^− 1^ and 1386 cm^− 1^ were slightly enhanced suggesting the hydrogen bonding interactions with ZnO nanoparticles.

Undoubtedly, incorporating tripolyphosphate (TPP) as a crosslinking agent into chitosan (CS) and zinc oxide (ZnO) nano-formulations results in efficient stabilization of the synthesized nano-formulation^90^. Of note, two distinctive peaks at 1134 cm^− 1^ and 885 cm^− 1^ corresponding to P=O stretching and the P–O–P bridge were primarily recorded in TPP spectrum (Fig. 5D). Regarding the FTIR spectrum of the *P. harmala*-TPP-CS-ZnO-NPs, the peak at 1685 cm^–1^ disappeared and the peak at 1594 cm^–1^ was shifted to 1559 cm^–1^ as well as a new peak around 1248 cm^− 1^, indicating the cross-linking of chitosan ammonium groups with the polyphosphoric group of TPP (N–O–P) via ionic gelatin (Fig. 5D).

### Morphology and additional DLS parameters

**Fig. 6:**
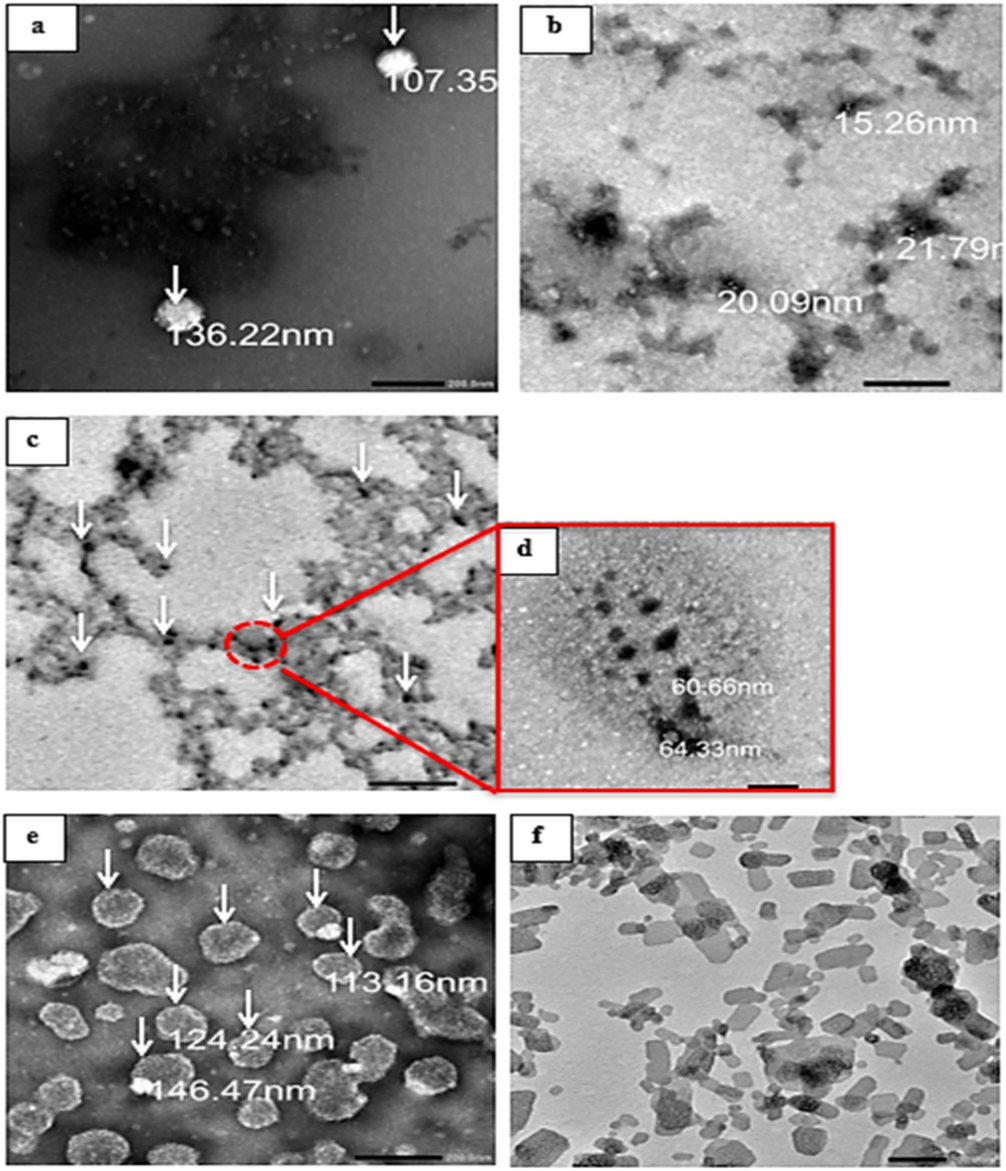
TEM photo-graphs of (**a**); *P. harmala*-CS-NPs (the arrow highlight the encapsulated herb inside NPs) and (**b**); *P. harmala*- ZnO NPs and (**c**); *P. harmala* -CS-ZnO NPs (the arrow highlight the ZnO NPs inside coated thickened chitosan matrix on ZnO NPs) and (**d**); a magnified single *P. harmala*-CS-ZnO Nps and (**e**); *P. harmala*-TPP-CS-ZnO NPs (the arrows highlight at the ZnO NP encapsulated within the chitosan nanoparticles (TPP-CS layers)) and (**f**); Blank ZnO NPs (without drug).

Figure 6 presents (TEM) images of various nanoparticle formulations loading *P. harmala* leaves extract with different nanoparticle systems. In general, the nanoparticles were uniform, homogenous distribution, small in size and their shape ranged from spherical to subspherical. The TEM photographs in Fig. 6 demonstrate the successful formulation and morphological differences between various nanoparticle systems used for encapsulating *P. harmala* extract as follows:

In Fig. 6a, the *P. harmala*-CS-NPs show relatively uniform spherical structures, with internal dark regions (highlighted by arrows) indicating successful encapsulation of the extract within the chitosan matrix.

*P. harmala*-ZnO NPs in (Fig. 6b) showed the ZnO nanoparticles loaded with the extract, displaying a granular morphology. The particles appear smaller and more uniform compared to chitosan-based systems.

*P. harmala*-CS-ZnO NPs in (Fig. 6c,d) reveals the hybrid nature of CS-ZnO NPs. The ZnO core is clearly embedded within a thicker chitosan coating, forming a core-shell structure that may enhance stability, reduce ZnO toxicity, and provide sustained drug release. Figure 6c showed the ZnO NPs are embedded in a chitosan matrix. The ZnO cores are visible as dark spots within a lighter polymeric coating. A magnified single *P. harmala*-CS-ZnO NP in (Fig. 6d) revealed the core-shell architecture, with a dense ZnO core surrounded by a thicker chitosan shell; for this reason, it is larger in size than *P. harmala* ZnO NPs in (Fig. 6b) due to coating with a chitosan matrix.

*P. harmala*-TPP-CS-ZnO NPs in (Fig. 6e) introduces TPP crosslinking into the CS-ZnO matrix. The presence of TPP improves the structural integrity of the chitosan shell and offers better encapsulation, as indicated by the more layered appearance and dense packaging. (Fig. 6e) showed the incorporating ZnO NPs into a crosslinked chitosan matrix using (TPP). The arrows highlight ZnO NPs encapsulated within multilayered chitosan and TPP structures. The Blank ZnO NPs in (Fig. 6f) showed the uncoated ZnO nanoparticles used as a control, appearing as small, crystalline, dispersed, and well-defined particles without any polymeric matrix.

These findings collectively demonstrate the successful manufacture of complex nanocarriers with the potential to enhance therapeutic delivery, particularly via improved encapsulation, particle stability, and release behavior.

The colloidal properties of various *P. harmala* leaves extract loaded nanocarriers were evaluated based on particle size, polydispersity index (PDI), zeta potential, entrapment efficiency (%EE) and drug loading capacity (%LC), which are critical indicators of nanoparticle stability, homogeneity, and drug-loading capacity. Table 3 showed the nano-formulations composition with acceptable physicochemical properties of four different Nano-formulations of *P. harmala* leaves extract loaded (CS NPs, ZnO NPs, CS-ZnO NPs, TPP-CS-ZnO NPs) and (blank ZnO NPs) such as; visual observation, particle size, PDI, Zeta potential, entrapment efficiency and loading capacity in (Table 4).

Preparing formulas with small particle size (PS) was one of the study’s primary objectives because the smaller particles could penetrate the skin membranes more deeply. The results (Table 4) showed that the PS measured by dynamic light scattering (DLS) of the studied four *P. harmala* leaves extract nanosystem formulae were arranged as follows in ascending order: ZnO NPs, CS-ZnO NPs, TPP-CS-ZnO NPs and CS NPs. This order is due to more layers being added by chitosan and TPP. These results are near and confirmed by the p. Size results obtained by TEM photographs in Fig. 6. However, the hydrodynamic diameter measured by DLS was slightly larger than the size obtained by TEM. The slightly larger size in DLS is attributed to the presence of the hydration layer surrounding the particles in solution. This difference, however, was small and within an acceptable range, indicating that the nanoparticles were well-dispersed with minimal aggregation in the medium.

Furthermore, the estimated PDI values are shown in (Table 4). All of the formulations demonstrated PDI values in the (0.32–0.56), which is an acceptable midrange^91^.

Zeta potential is a crucial metric for assessing both mono-dispersity and particle stability within a formula^92^. A strong enough double layer repulsion effect between the particles to stop them from aggregating is indicated by a high zeta potential, either positive or negative^93^. The ZP values specified in (Fig. S3) and Table 4 varied from − 15.1 to + 40.8 mV. The strong repulsive forces induced by the high ZP prevented the resultant nano-dispersion from aggregating^44,94^ especially in case of *P. harmala*-CS-ZnO NPs (+ 40.8). Also, zeta potential can predict interactions with surfaces such as with virus surface. All formulations were positive charge except for ZnO NPs (blank and *P. harmala* loaded ZnO NPs) were negative charges. The high (+ ve) charge of (*P. harmala*-CS-ZnO NPs) will show the best one, due to the presence of free amino groups of chitosan that give it a positive charge, which it may utilize for interaction with the negatively (− ve) charged viral surface, followed by *P. harmala*-TPP-CS-ZnO NPs (+ 24.2) and *P. harmala*-CS NPs (+ 19.2). Ideally, chitosan coating should provide positively charged particles but crosslinking with TPP (as in case of *P. harmala*-TPP-CS-ZnO NPs (+ 24.2) and *P. harmala*-CS NPs (+ 19.2) may be responsible for decreasing the positivity charge than coating with chitosan only without TPP (in case of *P. harmala*-CS-ZnO NPs). Other studies found similar behaviour following chitosan coating and TPP-induced crosslinking^95,96^.

It appears that chitosan can kill the virus directly by rupturing its protective envelope, based on the electrostatic interaction between its polycationic positive charge and the virus’s negatively charged surface^24^. In other hand, chitosan might also electrostatically attach to the cellular receptors and inhibit virus–host cell adsorption^97,98^. Additionally, it has been demonstrated that CS may efficiently enter cells and enhance the permeability of nanoparticles into cells^30^, making it a viable choice for creating antiviral medications that prevent viral multiplication within cells^99,100^. Therefore, CS can be utilized as a both drug delivery system and an antiviral agent while it can also decline drug cell toxicity.

The advancement of nanotechnology has made it possible to generate CS in a variety of morphologies using a variety of preparation techniques^24^. Among these morphologies, coating-another types of nanoparticles such as (metal NPs, lipids NPs,….etc) form can exhibit prominent properties in biomedical application due to the higher aspect ratio^24,101–103^. Since viruses multiply inside cells, this property may improve ZnO NPs’ antiviral efficacy and enhance CS’s penetration within the host cell^24^.

Both entrapment efficiency (%EE) and drug loading capacity (LC%) were calculated for each nano-system to enable a comprehensive evaluation of their drug delivery potential & antiviral performance.

The %EE values ranged from 52.8% to 81.7%, with the highest value observed in *P. harmal*-CS-ZnO NPs. Only two nanosystem formulas had %EE above 75% (Table 4), which are *P. harmal*-CS-ZnO NPs and *P. harmala*-CS NPs. These findings demonstrated the potential for successfully entrapping the *P. harmala* inside the CS NPs and CS-ZnO NPs. The results (Table 4) showed that the %EE of the four nanosystem formulae studied might be arranged as follows in ascending order: *P. harmala*-ZnO NPs, *P. harmala*-TPP-CS-ZnO NPs, *P. harmala*-CS NPs, and *P. harmala*- CS-ZnO NPs.

However, the %EE alone does not provide insight into how much drug is present relative to the total nanoparticles weight, which is critical for determining therapeutic dosage and minimizing carrier-related toxicity. Therefore, the %LC was also calculated, revealing a wide range between 4.8% and 33.3%. While *P. harmala* CS-NPs showed the highest %LC (33.3%) due to their low total mass and high drug content. The *P. harmala* –CS-ZnO NPs offered a balanced profile with high %EE (81.7%) and good %LC (6.81%).

According to previous results, the optimum nanoparticle formulation for *P. harmala* leaves extract loaded different nanosystems was *P. harmala* CS-ZnO NPs. Because, among the formulations, the *P. harmala* CS-ZnO NPs demonstrated the most favorable profile, with an average particle size of 73.06 ± 18.3 nm, a PDI of 0.40 ± 0.03, a highly stable zeta potential of + 40.8 ± 1.59 mV, and the highest entrapment efficiency of 81.7 ± 3.05%. These parameters fall within the ideal ranges for effective drug delivery systems, where particle sizes below 200 nm facilitate cellular uptake, PDI values below 0.5 suggest acceptable homogeneity, and zeta potentials above ± 30 mV indicate strong electrostatic stabilization^91,104^. In comparison, *P. harmala* CS NPs and *P. harmala* -TPP-CS-ZnO NPs also showed good %EE (75.0% and 66.8%, respectively), but had either larger particle sizes or lower colloidal stability. Notably, the *P. harmala* ZnO NPs exhibited the smallest particle size (29.10 ± 7.39 nm) yet had the lowest zeta potential (-4.83 ± 3.43 mV) and reduced %EE (52.8 ± 7.11%), rendering them less stable and less efficient in drug loading. Collectively, these findings establish the CS-ZnO nanocarrier as the optimal formulation, offering a well-balanced combination of nano-scale size, homogeneity, stability, and drug encapsulation capability suitable for enhanced therapeutic application of *P. harmala* leaves extract.

**Table 4:** Formulations colloidal properties of *P. harmala* leaves extract loaded different nanoparticles.

Formulation code	Visual observation*	Particle size ± SD (nm)	PDI ± SD	Zeta potential (mV ± SD)	%EE ± SD (%)	%LC ± SD (%)
Blank ZnO NPs	√	27.01 ± 6.19	0.47 ± 0.05	− 15.1 ± 1.29	–	–
*P. harmala*- CS NPs	√	145.5 ± 43.5	0.32 ± 0.03	+ 19.2 ± 8.95	75.0 ± 1.32	33.3% ± 0.59
*P. harmala*- ZnO NPs	√	29.10 ± 7.39	0.45 ± 0.05	− 4.83 ± 3.43	52.8 ± 7.11	4.8% ± 0.64
*P. harmala*-CS-ZnO NPs	√	73.06 ± 18.3	0.40 ± 0.03	+ 40.8 ± 1.59	81.7 ± 3.05	6.8% ± 0.25
*P. harmala*-TPP-CS-ZnO NPs	√	128.0 ± 16.9	0.56 ± 0.06	+ 24.2 ± 2.00	66.8 ± 0.81	5.45% ± 0.06

All formulations contain 1 mg *P. harmala* leaves extract each in 10 ml volume (CS solution 1:1 TPP solution), 0.1 mL 0.1% v/v Tween 80 stabilizers. CS, chitosan; TPP, tripolyphosphate; PDI, polydispersity index; %EE, % Entrapment Efficiency; %LC, Loading Capacity. * Opalescent colloidal dispersion (√).

### In vitro release rates for *P. harmala* leaves extract loaded Nano-formulations

**Fig. 7:**
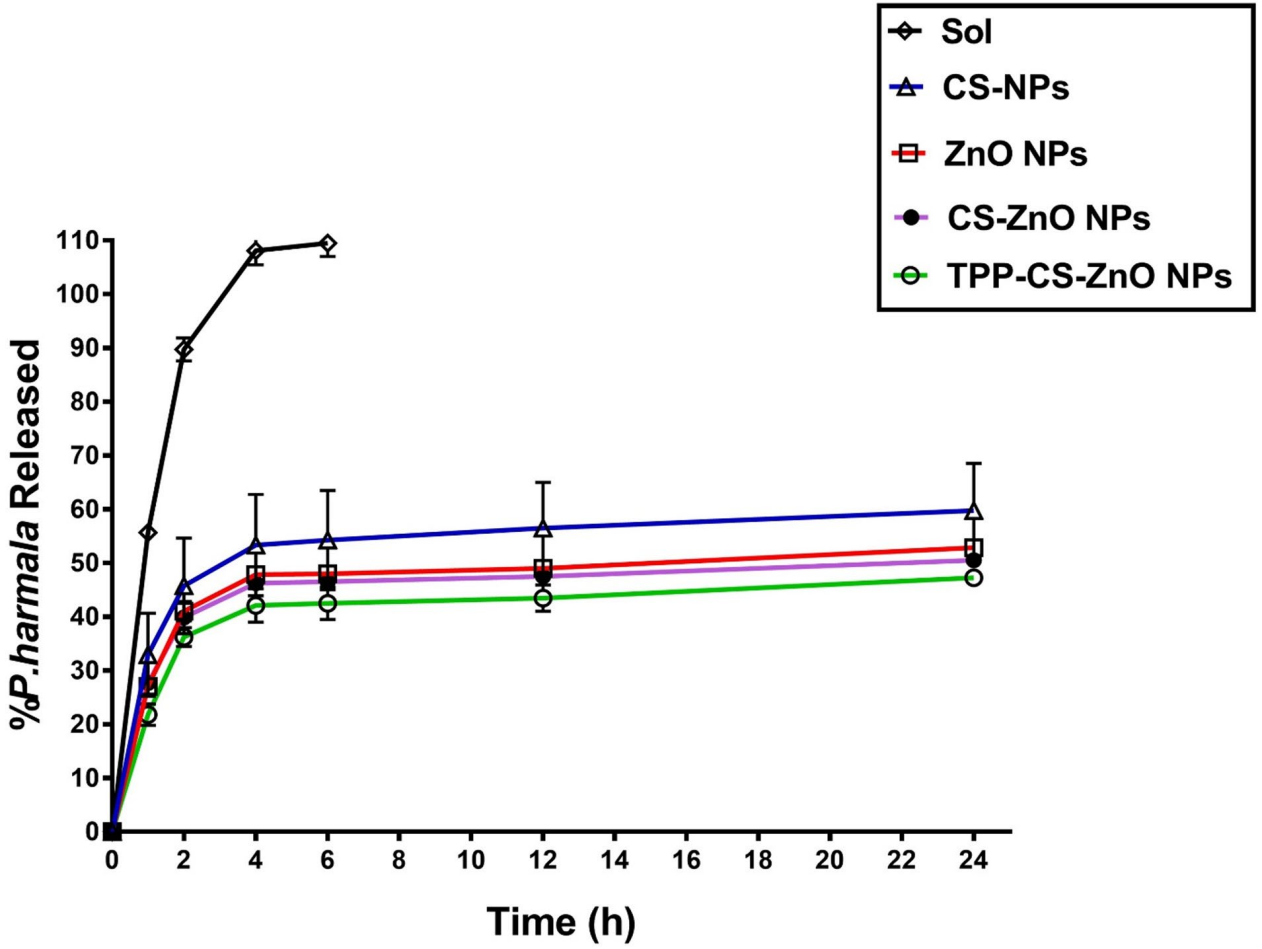
Cumulative In vitro release profiles of *P. harmala* leaves extract from four different NPs formulas in comparison to raw *P. harmala* leaves extract solution (Sol) in dissolution medium (PBS of pH 6.8).

The release profiles of *P. harmala* leaves extract formulations were evaluated in PBS (pH 6.8 at 37 °C) over a 24-h period using the dialysis-bag method. The release profiles of the four NPs formulations and pure *P. harmala* leaves extract solution are shown in Fig. 7. The (Fig. 7) displays the cumulative in vitro release profiles of a drug from five formulations: raw drug solution (Sol), chitosan nanoparticles (CS-NPs), zinc oxide nanoparticles (ZnO NPs), chitosan-coated zinc oxide nanoparticles (CS-ZnO NPs), and TPP cross-linked chitosan-coated zinc oxide nanoparticles (TPP-CS-ZnO NPs) over 24 h.

As expected, the raw *P. harmala* leaves extract solution demonstrated a rapid and complete release (~ 100%) within the initial 2 h, attributed to the lack of a delivery matrix and the immediate diffusion of the drug into the release medium.

The release rates among the nanoparticulate formulations exhibited considerable variation.

- *P. harmala* CS-NPs showed the highest release among the nanoformulations, with ~ 60% cumulative release at 24 h. This is likely due to the hydrophilic and porous nature of chitosan, which facilitates faster drug diffusion.
- *P. harmala* ZnO NPs and *P. harmala* CS-ZnO NPs demonstrated intermediate release behaviors (~ 50–55%), where the chitosan coating moderately slowed drug diffusion through additional matrix complexity.
- *P. harmala* TPP-CS-ZnO NPs exhibited the lowest and most sustained release profile, with only ~ 45% release over 24 h. The tight, cross-linked structure formed by tripolyphosphate (TPP) interaction with (CS) creates a dense polymer network, effectively delaying drug release.

Based on the cumulative in vitro release profile, *P. harmala* TPP-CS-ZnO NPs clearly demonstrate a slow and sustained drug release profile. This is attributed to:

1. Ionic cross-linking between the negatively charged TPP and positively charged CS, which reduces matrix porosity and drug mobility, which creates a tighter matrix that retards drug diffusion^105,106^.
2. Moreover, the hybrid structure (Layered nanostructure) that includes ZnO NPs as a core, CS as a coating, and TPP as a stabilizer, enhancing structural integrity, which further contributing to the controlled release profile^106–108^.
3. Electrostatic and hydrogen bonding interactions between the *P.harmala* leaves extract drug and the polymeric network, contributing to delayed release^46^.

These results are consistent with earlier research showing that TPP-cross linked CS NPs considerably prolonged drug release^46,105,106,108^, where, Agnihotri et al. (2004) reported that TPP-cross linked CS matrices show delayed drug diffusion due to enhanced network density. Also, Nafee et al. (2009) and Calvo et al. (1997) confirmed that TPP and ZnO-chitosan combinations improve the stability and release control of nano-drug carriers. This sustained release behavior is highly beneficial for prolonged therapeutic action and reduced dosing frequency, which is advantageous in drug delivery systems.

The tested four nano-formulas’ released *P. harmala* leaves extract showed that; all nano-formulations have slower release than pure extracts alone. It’s possible that the complexation between *P. harmala* leaves extract and the CS polymer (in case of CS NPS), the ZnO (in case of ZnO Nps), both the CS and ZnO (in case of CS-ZnO NPs) and TPP, CS and ZnO (in case of TPP-CS-ZnO NPs) took some time to dissolve and let the drug diffuse outside the nanoparticles, which is why all nano-formulations released considerably more slowly than pure extract alone. The gradual release of the active ingredients from the four nanosystems is proof that the drug is uniformly entrapped throughout the systems depending on the number of layers and components of each type of nano-formula which is considered as a barrier for drug to be dissolved and diffuse outside the nanoparticles. Consequently, the drug release of the studied formulae might be arranged as follows in ascending order: *P. harmala*-TPP-CS-ZnO NPs dispersions, *P. harmala*-CS-ZnO NPs dispersions, *P. harmala*- ZnO NPs dispersions, *P. harmala*-CS NPs dispersions, and pure *P. harmala* solution. In this case, the four *P. harmala* leaves extract nano-formulations release was sustained in comparison to pure raw extract in solution form affected by the entrapment component layers of drug inside nano-formulations & solubility of the drug.

The four nano-formulas dispersions for *P. harmala* leaves extract demonstrated a sustained or prolonged release over 24 h in the in vitro release profiles when compared to pure extract in solution form (Fig. 7). The four *P. harmala* leaves extract nanosystems exhibited a gradual slow release of *P. harmala* leaves extract ingredients, indicating that the drug was uniformly entrapped throughout the systems. Furthermore, the cumulative in vitro release rate of the four nano-formulations decreased with the increasing nanoparticles coating layers (component ingredients). At the 24 h drug cumulative release rate was 59.72 ± 8.78, 52.85 ± 1.40%, 50.52 ± 0.31 and 47.26 ± 0.48 for *P. harmala*-CS NPs, *P. harmala*-ZnO NPs, *P. harmala*-CS-ZnO NPs and *P. harmala*-TPP-CS-ZnO NPs respectively.

The biphasic release behavior of the four *P. harmala* leaves extract nanoparticle formulas (CS, ZnO, CS-ZnO and TPP-CS-ZnO) is also readily observable; these release profiles show a relatively quick release within the first one hour (21.8–27%), which is followed by a more delayed release pattern. One possible explanation for the first burst release from drug loaded with NPs is that the free drug molecules attached to the NPs surface released quickly, whereas the entrapped drug molecules released more slowly^109^.

### Dissolution profile comparison

After performing the in vitro release test, a mathematical comparison is calculated using the difference factor (*f1*) and similarity factor (*f2*). The FDA guidelines stated that *f1* values up to 15 (0–15) and *f2* values greater than 50 (50–100) ensure sameness or equivalence of test product to the reference product^110^. For each of the four prepared *P. harmala* leaves extract nano-formulations, (*f1*) and (*f2*) were computed^111-113^ in comparison to the *P. harmala* leaves extract solution (Table 5). The results of the two parameters (*f1* over 15 and *f2* below 50) showed that, in terms of release profile, none of the *P. harmala* leaves extract nano-formulations were similar to the raw *P. harmala* leaves extract solution.

Statistical analysis was used to compute a second dissolving parameter, percent dissolution efficiency ( %DE_24h_)^114,115^. The computed statistics support the earlier findings; Table 5 displays a significant variation in %DE between all four *P. harmala* leaves extract nano-formulations and *P. harmala* leaves extract solution.

**Table 5:** Difference factor (*f1*) and similarity factor (*f2*) and percent dissolution efficiency (% D.E) calculated values for tested *P. harmala* leaves extract nano-formulas compared *P. harmala* leaves extract solution as a reference product.

Formula code	*f1* value	*f2* value	%D.E_24h_
*P. harmala* sol	0^a^	100^a^	103.9 ± 19.5
*P. harmala*- CS NPs	48^b^	19^b^	53.82 ± 8.50^c^
*P. harmala*- ZnO NPs	54^b^	16^b^	47.25 ± 1.15^c^
*P. harmala*-CS-ZnO NPs	56^b^	16^b^	45.67 ± 0.47^c^
*P. harmala*-TPP-CS-ZnO NPs	60^b^	14^b^	41.86 ± 1.84^c^

^a^Similar, ^b^different, ^c^Statistically significant at (*p* ≤ 0.05), Statistical analysis was done using one-way ANOVA followed by Student–Newman–Keuls multiple comparison test.

### In vitro antiviral study

#### Cytotoxicity concentration 50 (CC_50_) of *P. harmala* leaves extract and its nano-formulations

At first, the cytotoxicity of the *P. harmala* leaves extract and the synthesized nano-formulations was evaluated using the MTT assay on Vero cell lines to ascertain safe, effective dosages of tested samples for the antiviral activity and prevent confusion between cytotoxicity and antiviral bioactivity. As presented in Fig. S4 and Table 6, our result dissected that both *P. harmala* leaves extract and chitosan nanoparticles loaded with harmala extract are generally considered biocompatible with higher cytotoxicity concentrations (CC_50_) equal 407.6 µg/ml and 575.4 µg/ml, respectively. In contrast, ZnO nanoparticles have demonstrated cytotoxic action with CC_50_ value of 92.95 µg/ml which moderately declined by incorporating *P. harmala* leaves extract due to the antioxidant capacity of harmala extract bioactive compounds. However, when the ZnO nanoparticles were encapsulated or modified with chitosan, the CC_50_ of established nano-formulation was evidently shifted to a higher CC_50_ value (271.4 µg/ml) (Table 6).

#### Plaque reduction assay for *P. harmala* leaves extract and its nano-formulations

*P. harmala* leaves extract had medium antiviral activity against Herpes simplex type one, with Inhibitory Concentration (IC_50_) = 23.91 µg/ml. The antiviral activity of different *P. harmala* leaves extract nano-formulations against (HSV-1), was evaluated by the reduction in plaque-forming units (PFU/mL) after treatment. The antiviral efficacy is presented as viral inhibition percentage, calculated against a constant viral control value of 1.85 × 10^7^ PFU/mL.

The results of the Plaque Reduction Assay are summarized in Table 6, which shows the viral count after treatment and the corresponding percentage of viral inhibition for each formulation at different concentrations (50, 25, 12.5, and 6.25 µg/mL). There are six formulas, four of them are tested different *P. harmala* leaves extract nano-formulas in comparison to raw *P. harmala* leaves extract (without nano-formula) and blank ZnO nanoparticles.

**Table 6:** Antiviral activity for *P. harmala* leaves extract and its nano-formulations by plaque reduction assay method.

Sample code	CC_50_ (µg/mL)	Conc. (µg/mL)	Virus control (PFU/mL)	Viral count after treatment (PFU/mL)	Viral inhibition (%)
*P. harmala*-CS NPs		50	1.85 × 10^7^	1.0 × 10^7^	45.9
	575.4	25		1.15 × 10^7^	37.8
		12.5		1.3 × 10^7^	29.7
		6.25		1.5 × 10^7^	18.9
*P. harmala*-ZnO NPs		50		0.95 × 10^7^	48.6
	165.75	25		1.1 × 10^7^	40.5
		12.5		1.25 × 10^7^	32.4
		6.25		1.35 × 10^7^	27
*P. harmala*-CS-ZnO NPs		50		0.85 × 10^7^	54.1
	271.4	25		0.9 × 10^7^	51.4
		12.5		1.0 × 10^7^	44.9
		6.25		1.2 × 10^7^	35.1
*P. harmala*-TPP-CS-ZnO NPs		50		0.9 × 10^7^	51.4
	240.5	25		1.1 × 10^7^	40.5
		12.5		1.2 × 10^7^	35.1
		6.25		1.4 × 10^7^	24.3
*P. harmala* extract		50		1.35 × 10^7^	27
	407.6	25		1.4 × 10^7^	24.3
		12.5		1.45 × 10^7^	21.6
		6.25		1.5 × 10^7^	18.9
Blank ZnO NPs	92.95	50		1.5 × 10^7^	24.3
		25		1.55 × 10^7^	18.9
		12.5		1.6 × 10^7^	13.5
		6.25		1.7 × 10^7^	8.1

### Antiviral activity

All formulations exhibited varied levels of antiviral efficacy against HSV-1, according to the results, with the maximum inhibition shown at the highest concentration (50 µg/mL). *P. harmala*-CS-ZnO NPs formula showed the strongest antiviral efficacy, with a maximum virus inhibition of 54.1% (at a drug concentration of 50 µg/mL). Significant action was also demonstrated by *P. harmala*- TPP-CS-ZnO NPs formula, which attained 51.4% inhibition at the same drug concentration.

The descending order of antiviral activity was as follows: The *P. harmala*-CS-ZnO NPs at 50 µg/mL showed the highest viral inhibition of 54.1%, with a post-treatment viral count of 0.85 × 10^7^ PFU/mL. This is followed closely by; *P. harmala*-TPP-CS-ZnO NPs (with 51.4% inhibition), *P. harmala*-ZnO NPs (with 48.6% inhibition) and *P. harmala*-CS NPs (with 45.9% inhibition) in comparison to pure *P. harmala* leaves extract with 27% inhibition and blank ZnO NPs with 24.3% inhibition.

### Concentration-dependent effect

A clear dose-dependent antiviral effect is observed across all formulations, with the highest inhibition at 50 µg/mL concentration, which gradually decreases with lower concentrations. The inhibitory effects were less noticeable at lower drug concentrations (e.g., 6.25 µg/mL), demonstrating the antiviral activity’s dose-dependent nature. Only 27% of the inhibition was obtained by the crude drug (*P. harmala* leaves extract), suggesting that the prepared nanoparticle preparations were superior to the raw substance.

### Comparison with crude drug (*P. harmala* leaves extract)

The pure *P. harmala* leaves extract without loading nano-formula, showed only 27% inhibition against HSV-1 at 50 µg/mL concentration while the other four nano-formulas showed around doubled the effect especially for *P. harmala*-CS-ZnO NPs formula. This enhanced effect is likely due to the synergistic antiviral properties of *P. harmala* bioactive compounds, chitosan, and ZnO nanoparticles, which together improve cellular uptake, viral inhibition, and bioavailability.

The blank ZnO NPs exhibited antiviral activity, with a maximum inhibition of only 24.3%, indicating that the choice of ZnO as a metal nanoparticle for a drug carrier was a good choice to increase the antiviral activity of *P. harmala* leaves extract.

Previous studies have shown that chitosan (CS) has antibacterial capabilities through the disruption of microbial membranes^116^. As a result, the chitosan action would work in conjunction with the *P. harmala* drug to treat herpes simplex type 1 infection like an antimicrobial agent.

Numerous studies support our finding that; nanoparticle formulations, particularly those involving metal oxides and chitosan, significantly enhance antiviral efficacy of plant extracts by improving drug stability, targeted delivery, and controlled release^20,24,117^.

Finally, the enhanced antiviral efficacy of the *P. harmala*–CS–ZnO NPs can be attributed to the synergistic interaction among its bioactive components. First, *P. harmala* leaves extract is rich in β-carboline alkaloids such as harmine and harmaline, which have demonstrated significant antiviral properties against a variety of DNA and RNA viruses^118^. Second, chitosan, a biocompatible polysaccharide, enhances cellular uptake through its mucoadhesive and polycationic characteristics, facilitating efficient transport of encapsulated compounds across cellular membranes^119^. Finally, zinc oxide nanoparticles possess inherent antiviral activity, largely through the production of reactive oxygen species and disruption of viral entry mechanisms^120^. The integration of these three components into a unified nanosystem results in a multi-targeted antiviral strategy, enhancing potency beyond that of the individual elements. This finding aligns with previous research demonstrating that multifunctional nanoparticle-based systems can significantly improve the stability, delivery, and therapeutic performance of plant-derived antiviral agents^121,122^.

Therefore, the *P. harmala*-CS-ZnO NPs formulation is showing a promising formulation as a potential therapeutic modality for HSV-1 infections.

### Mode of action for *P. harmala*-CS-ZnO NPs formula against herpes simplex virus type 1 (HSV-1)

Complementarily, with a scope to strengthen biological interpretability and relevance, the mode of action study of *P. harmala*-CS-ZnO NPs formula with the most promising observations against HSV-1 was conducted. The gathered data displayed in Table S4 revealed that the effective anti-HSV-1 effect for *P. harmala*-CS-ZnO NPs formula (50 µg/ml) was mainly due to virucidal and adsorption inhibitory actions with viral inhibition values of 31.5% and 16%, respectively. Further, our findings demonstrated replication inhibition mechanism for *P. harmala*-CS-ZnO NPs formula by 7.4%.

To sum up, *P. harmala*-CS-ZnO NPs formula can act on different viral phases by effectively interfering with the virus attachment proteins and inactivating extracellular viral particles (virions) via damaging the viral protein capsid or the viral genome.

### Investigated correlation

**Fig. 8:**
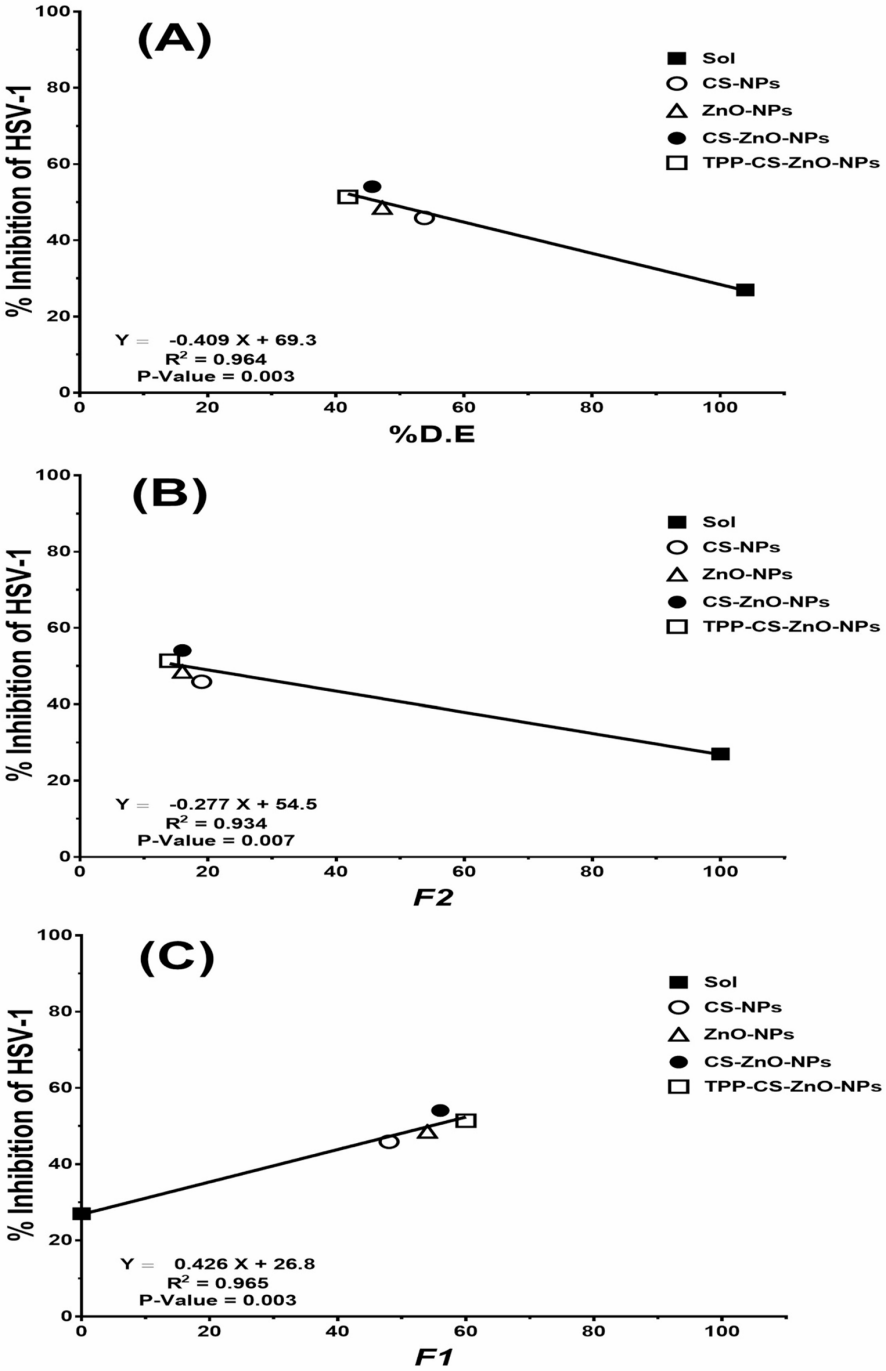
Correlation between % Inhibition of HSV-1 and (**A**): % D.E _24 h_, (**B**): *f2*, (**C**): *f1*.

A quantitative relationship between in vitro pharmaceutical data and in vitro microbiological characteristics was investigated. Dissolution Efficiency, (% D.E) and the % inhibition of HSV-1 revealed a significant correlation coefficient R^2^ = 0.964 with (*p-value* = 0.003) (Fig. 8).

Furthermore, a correlation between the *f1* or *f2* against % inhibition of HSV-1 revealed a significant value of R^2^ = 0.965 and 0.934 with a *p-value* of 0.003 and 0.007 for *f1* and *f2* respectively (Fig. 8).

## Conclusion

The current study offered an integrative workflow featuring UPLC-MS based chemical profiling, network pharmacology analysis, nanoscience, and experimental validation to objectively pinpoint the multi-scale efficacy mechanisms of *P. harmala* bioactive compounds against HSV-1. The network pharmacology analysis predicted MAPK 1, SRC, EGFR and JAK1 as the top HSV-1 genes co-regulated by *P. harmala* bioactive compounds primarily harmine, peganone 2, vasicine, and coumaric acid and highly correlated with MAPK, PI3K-Akt, JAK-STAT signalling pathways which are directly implicated in HSV-1 pathogenesis. Complementarily, different nano-formulations based on Zinc oxide and chitosan were established where their antiviral effects were experimentally assessed using plaque reduction assay. Practically speaking, the *P. harmala* CS-ZnO NPs afforded the most promising observations manifested by optimal balance of physicochemical properties, sustained drug release, and potent antiviral activity (54.1% virus inhibition at 50 µg/mL). The enhanced antiviral efficacy of the *P. harmala*-CS-ZnO NPs can be attributed to the synergistic interaction among *P. harmala*, chitosan and ZnO NPs components. These findings support its potential as a highly effective nanocarrier system for enhancing the therapeutic efficacy of *P. harmala* leaves extract in antiviral applications.

Collectively, these findings not only reinforce the therapeutic value of *P. harmala* leaves extract but also support its continued investigation as a source of novel, plant-based antiviral agents. Nevertheless, further mechanistic explanations and enough evidence should be investigated to bolster the biological relevance.

## Supplementary Information

Below is the link to the electronic supplementary material.

Supplementary Material 1

## Data Availability

Data is provided within the manuscript or supplementary information files.
