# Supplementary material for: Comparison of Peganum harmala L. leaves extract nanoformulations against herpes simplex virus type 1 guided by network pharmacology analysis
Source: Sci Rep. 2025 Nov 18;15:40395. doi: 10.1038/s41598-025-24155-9 (PMC12627805; doi:10.1038/s41598-025-24155-9)
Supplement: Supplementary file 1 — Supplementary Material 1 [file 41598_2025_24155_MOESM1_ESM.docx]

***UPLC-ESI-QqQ-MS/MS analysis of Peganum harmala***

Chemical proﬁling of *P. harmala* leaves extract was performed using UPLC XEVO TQD triple quadruple instrument Waters Corporation, Milford, MA01757 U.S.A. Chromatographic separation was implemented on Waters Acquity UPLC BEH C18 column (50 mm × 2.1 mm ID × 1.7 μm particle size) set up at 30^°^C. The biphasic mobile phase comprised of acidiﬁed ultrapure water (0.1% formic acid) (Phase A) and acidiﬁed acetonitrile (0.1% formic acid) (Phase B) was gradient eluted at a ﬂow rate of 0.2 ml/ min and programmed as follows: 0–3 min, 5–20% B; 3–6 min, 20–60% B; 6–12 min, 60- 60% B; 12–15 min, 60–80% B; 15–20 min, 80–100% B; 20–25 min, 100- 100% B with a post-run of 5 min to equilibrate the system. The optimized parameters for ESI interface operated in positive or negative with full scan function from m/z 50_~_1200 were as follows: capillary voltage of 3 kV, cone voltage; 35 V, the ion source temperature was 150◦C, the nebulizer (nitrogen gas) pressure was 35 psi, drying and sheath gas (N_2_) temperature was 440◦C and 350◦C, respectively. The drying and sheath gas ﬂows were applied at 900 L/h and 50 L/h, respectively. The scan time and interscan delay were set to 0.4 s and 0.1 s, respectively and the analytical run time was extended to 30 min. In MS^E^ mode, 3 collision energies of 10 eV, 20 eV and 40 eV were conducted.

Of note, 5 µl from each examined sample were pooled to generate a quality control (QC) sample for judging the stability and robustness of the analytical platform.

**MTT cytotoxicity assay for *P. harmala* leaves extract and its nano-formulations**

To determine the half-maximal cytotoxic concentration (CC_50_) ^[49]^, stock solutions of the test compounds were prepared in 10% DMSO in ddH_2_O and further diluted to working solutions with DMEM. The cytotoxic activity of the extracts was tested in VERO-E6 cells using the 3-(4,5-dimethylthiazol-2-yl)-2,5-diphenyltetrazolium bromide (MTT) method with minor modifications. Briefly, the cells were seeded in 96 plates (100 µl/well at a density of 3×10^5^ cells/ml) and incubated for 24 h at 37 °C in 5% CO_2_. After 24 h, cells were treated with various concentrations of the tested compounds in triplicate. 24 hours later, the supernatant was discarded, and the cell monolayers were washed with sterile 1x phosphate-buffered saline (PBS) three times. MTT solution (20 µl of a 5 mg/ml stock solution) was then added to each well and incubated at 37 °C for 4 hours, followed by medium aspiration. In each well, the former formazan crystals were dissolved with 200 µl of acidified isopropanol (0.04 M HCl in absolute isopropanol = 0.073 ml HCL in 50 ml isopropanol). Absorbance of formazan solutions was measured at λ _max_ 540 nm with 620 nm as a reference wavelength using a multi-well plate reader. The percentage of cytotoxicity compared to the untreated cells was determined with the following equation.

The plot of % cytotoxicity versus sample concentration was used to calculate the concentration that exhibited 50% cytotoxicity (CC_50_) ^[49]^.

**% Cytotoxicity=(Absorbance of cells without treatment-Absorbance of cells with treatment)X 100**

**(Absorbance of cells without treatment)**

**Table S1: The 25 candidate compounds from the *P. harmala* leaves extract and their ADME screening information**

| **Compound name** | **mol MW** | **donorHB** | **accptHB** | **QPlogP** | **OB (%)** |
| --- | --- | --- | --- | --- | --- |
| Gamma amino butyric acid (GABA) | 103.2 | 6 | 4.7 | -0.457 | 33.288 |
| Citric acid | 192.2 | 1 | 5 | -1.113 | 78.924 |
| Protocatechuic acid | 154.3 | 6 | 2.25 | -0.02 | 70.516 |
| Hydroxy benzoic acid | 138.3 | 6 | 2.75 | 0.578 | 64.071 |
| Coumaric acid | 165.3 | 2 | 2 | -0.125 | 73 |
| Peganone1 | 284.3 | 1 | 4 | 0.819 | 85 |
| Pegamine | 204.3 | 2 | 4 | 0.884 | 87 |
| Harmine | 212.2 | 2 | 2 | -2.54 | 100 |
| Vasicine (peganine) | 188.3 | 1 | 4 | 0.819 | 85.3 |
| Peganone2 | 268.2 | 2 | 4 | 0.889 | 86.7 |
| Pegamine dimer | 410.3 | 2 | 4 | 0.884 | 87 |
| Acacetin-O-rutinoside (Linarin) | 592.3 | 2 | 3 | 1.596 | 100 |
| Acacetin | 284.3 | 2 | 3 | 1.596 | 100 |
| Harman | 182.3 | 2 | 2 | 1.596 | 88 |
| Harmol | 198.2 | 2 | 3 | 1.567 | 100 |
| Harmalidine | 254.3 | 2 | 2 | 1.34 | 100 |
| Rosmanol | 348.2 | 2 | 3.5 | 4.838 | 92.013 |
| Palmitic acid | 256.2 | 6 | 3.7 | 4.256 | 88.722 |
| Stearidonic acid | 276.3 | 4 | 2 | 3.056 | 88.19 |
| Hydroxy linolenic acid | 294.3 | 4 | 3.7 | 4.197 | 88.926 |
| δ-Tocotrienol | 396.3 | 2 | 5 | 0.086 | 77.4 |
| Hydroxy linoleic acid | 296.3 | 4 | 3.7 | 4.211 | 92.888 |
| Linolenic acid | 278.3 | 4 | 2 | 5.236 | 87.243 |
| β-Tocopherol | 416.3 | 2 | 7 | -0.24 | 78.4 |
| β-Sitosterol | 414.2 | 2 | 4 | 0.677 | 87.2 |

*The acceptable ADME values are molecular weight (MW)≤500, hydrogen bond acceptors (Hacc≤10), hydrogen bond donors (Hdon ≤5), and octanol-water partition coefficient lesser than five (LogP≤5)

*Favorable oral bioavailability (OB) score ≥ 30%

**Table S2: Potential protein targets of *Peganum harmala* leaves extract derived constituents**

| **Uniport ID** | **Short name of protein** | **Full name of protein** |
| --- | --- | --- |
| [P05121](https://www.uniprot.org/uniprotkb/P05121/entry) | SERPINE1 | Plasminogen activator inhibitor 1 |
| [P06239](https://www.uniprot.org/uniprotkb/P06239/entry) | [LCK](http://www.genecards.org/cgi-bin/carddisp.pl?gene=LCK&search=LCK) | Tyrosine-protein kinase Lck |
| [P17252](https://www.uniprot.org/uniprotkb/P17252/entry) | [PRKCA](http://www.genecards.org/cgi-bin/carddisp.pl?gene=PRKCA&search=PRKCA) | Protein kinase C alpha type |
| [P12931](https://www.uniprot.org/uniprotkb/P12931/entry) | [SRC](http://www.genecards.org/cgi-bin/carddisp.pl?gene=SRC&search=SRC) | Proto-oncogene tyrosine-protein kinase Src |
| [P09874](https://www.uniprot.org/uniprotkb/P09874/entry) | [PARP1](http://www.genecards.org/cgi-bin/carddisp.pl?gene=PARP1&search=PARP1) | Poly [ADP-ribose] polymerase 1 |
| [Q00537](https://www.uniprot.org/uniprotkb/Q00537/entry) | [CDK2](http://www.genecards.org/cgi-bin/carddisp.pl?gene=CDK2&search=CDK2) | Cyclin-dependent kinase 17 |
| [O00206](https://www.uniprot.org/uniprotkb/O00206/entry) | [TLR4](http://zinc15.docking.org/genes/TLR4) | Toll-like receptor 4 |
| [P14780](https://www.uniprot.org/uniprotkb/P14780/entry) | [MMP9](http://www.genecards.org/cgi-bin/carddisp.pl?gene=MMP9&search=MMP9) | Matrix metalloproteinase-9 |
| [O23507](https://www.uniprot.org/uniprotkb/O23507/entry) | [MMP1](http://www.genecards.org/cgi-bin/carddisp.pl?gene=MMP1&search=MMP1) | Matrix metalloproteinase-1 |
| [P08253](https://www.uniprot.org/uniprotkb/P08253/entry) | [MMP2](http://www.genecards.org/cgi-bin/carddisp.pl?gene=MMP1&search=MMP1) | Matrix metalloproteinase-2 |
| [E7BSV0](https://www.uniprot.org/uniprotkb/E7BSV0/entry) | [EGFR](http://www.genecards.org/cgi-bin/carddisp.pl?gene=EGFR&search=EGFR) | Receptor protein-tyrosine kinase |
| [P23219](https://www.uniprot.org/uniprotkb/P23219/entry) | [TYR](http://www.genecards.org/cgi-bin/carddisp.pl?gene=TYR&search=TYR) | Tyrosinase |
| [P09417](https://www.uniprot.org/uniprot/P09417) | [PTGS2](http://www.genecards.org/cgi-bin/carddisp.pl?gene=PTGS2&search=PTGS2) | Prostaglandin G/H synthase 2 |
| [P31749](https://www.uniprot.org/uniprot/P31749) | AKT1 | RAC-alpha serine/threonine-protein kinase |
| [Q9NRD5](https://www.uniprot.org/uniprot/Q9NRD5) | PRKCA | PRKCA-binding protein |
| [P15692](https://www.uniprot.org/uniprot/P15692) | VEGFA | Vascular endothelial growth factor A |
| [O14788](https://www.uniprot.org/uniprotkb/O14788/entry) | [TNF](http://www.genecards.org/cgi-bin/carddisp.pl?gene=TNF&search=TNF) | Tumor necrosis factor |
| [P08887](https://www.uniprot.org/uniprotkb/P08887/entry) | [IL6](http://www.genecards.org/cgi-bin/carddisp.pl?gene=IL2&search=IL2) | Interleukin-6 |
| [Q9NR96](https://www.uniprot.org/uniprotkb/Q9NR96/entry) | [TLR9](http://www.genecards.org/cgi-bin/carddisp.pl?gene=TLR9&search=TLR9) | Toll-like receptor 9 |
| [P09917](https://www.uniprot.org/uniprotkb/P09917/entry) | [ALOX5](http://www.genecards.org/cgi-bin/carddisp.pl?gene=ALOX5&search=ALOX5) | Arachidonate 5-lipoxygenase |
| [P52333](https://www.uniprot.org/uniprotkb/P52333/entry) | [JAK3](http://www.genecards.org/cgi-bin/carddisp.pl?gene=JAK3&search=JAK3) | Tyrosine-protein kinase JAK3 |
| [[O43318](https://www.uniprot.org/uniprotkb/O43318/entry)](https://www.uniprot.org/uniprot/P43088) | [MAPK8](http://www.genecards.org/cgi-bin/carddisp.pl?gene=MAPK8&search=MAPK8) | mitogen-activated protein kinase 8 |
| [P31751](https://www.uniprot.org/uniprotkb/P31751/entry) | AKT2 | RAC-beta serine/threonine-protein kinase |
| [O00141](https://www.uniprot.org/uniprotkb/O00141/entry) | SGK1 | Serine/threonine-protein kinase Sgk1 |
| [[[P45452](https://www.uniprot.org/uniprotkb/P45452/entry)](https://www.uniprot.org/uniprot/P43116)](https://www.uniprot.org/uniprot/Q62053) | [MMP13](http://www.genecards.org/cgi-bin/carddisp.pl?gene=MMP13&search=MMP13) | Collagenase 3 |
| [[P39900](https://www.uniprot.org/uniprotkb/P39900/entry)](https://www.uniprot.org/uniprot/Q9HBW0) | [MMP12](http://www.genecards.org/cgi-bin/carddisp.pl?gene=MMP12&search=MMP12) | Macrophage metalloelastase |
| [[P22894](https://www.uniprot.org/uniprotkb/P22894/entry)](https://www.uniprot.org/uniprot/Q9UBY5) | [MMP8](http://www.genecards.org/cgi-bin/carddisp.pl?gene=MMP8&search=MMP8) | Neutrophil collagenase |
| [P50281](https://www.uniprot.org/uniprotkb/P50281/entry) | [MMP14](http://www.genecards.org/cgi-bin/carddisp.pl?gene=MMP14&search=MMP14) | Matrix metalloproteinase-14 |
| [P03956](https://www.uniprot.org/uniprotkb/P03956/entry) | [MMP1](http://www.genecards.org/cgi-bin/carddisp.pl?gene=MMP14&search=MMP14) | Interstitial collagenase |
| [P14780](https://www.uniprot.org/uniprotkb/P14780/entry) | [MMP9](http://www.genecards.org/cgi-bin/carddisp.pl?gene=MMP1&search=MMP1) | Matrix metalloproteinase-9 |
| [[P23458](https://www.uniprot.org/uniprotkb/P23458/entry)](https://www.uniprot.org/uniprot/P04035) | [JAK1](http://www.genecards.org/cgi-bin/carddisp.pl?gene=JAK1&search=JAK1) | Tyrosine-protein kinase JAK1 |
| [Q07869](https://www.uniprot.org/uniprot/Q07869) | PPARA | Peroxisome proliferator-activated receptor alpha |
| [O60603](https://www.uniprot.org/uniprot/O60603) | TLR2 | Toll-like receptor 2 |
| [O60674](https://www.uniprot.org/uniprotkb/O60674/entry) | [JAK2](http://www.genecards.org/cgi-bin/carddisp.pl?gene=JAK2&search=JAK2) | Tyrosine-protein kinase JAK2 |
| [Q16539](https://www.uniprot.org/uniprotkb/Q16539/entry) | [MAPK14](http://www.genecards.org/cgi-bin/carddisp.pl?gene=MAPK14&search=MAPK14) | Mitogen-activated protein kinase 14 |
| [P23443](https://www.uniprot.org/uniprotkb/P23443/entry) | [RPS6KB1](http://www.genecards.org/cgi-bin/carddisp.pl?gene=RPS6KB1&search=RPS6KB1) | Ribosomal protein S6 kinase beta-1 |
| [P28907](https://www.uniprot.org/uniprotkb/P28907/entry) | [CD38](http://www.genecards.org/cgi-bin/carddisp.pl?gene=CD38&search=CD38) | ADP-ribosyl cyclase/cyclic ADP-ribose hydrolase 1 |
| [Q16644](https://www.uniprot.org/uniprotkb/Q16644/entry) | [MAPK3](http://www.genecards.org/cgi-bin/carddisp.pl?gene=MAPK3&search=MAPK3) | MAP kinase-activated protein kinase 3 |
| [P60953](https://www.uniprot.org/uniprotkb/P60953/entry) | CDC42 | Cell division control protein 42 homolog |
| [O15111](https://www.uniprot.org/uniprotkb/O15111/entry) | [CHUK](http://www.genecards.org/cgi-bin/carddisp.pl?gene=CHUK&search=CHUK) | Inhibitor of nuclear factor kappa-B kinase subunit alpha |
| [P45984](https://www.uniprot.org/uniprotkb/P45984/entry) | [MAPK9](http://www.genecards.org/cgi-bin/carddisp.pl?gene=MAPK9&search=MAPK9) | Mitogen-activated protein kinase 9 |
| [Q9NWZ3](https://www.uniprot.org/uniprotkb/Q9NWZ3/entry) | [IRAK4](http://www.genecards.org/cgi-bin/carddisp.pl?gene=IRAK4&search=IRAK4) | Interleukin-1 receptor-associated kinase 4 |
| [Q16539](https://www.uniprot.org/uniprotkb/Q16539/entry) | [MAPK14](http://www.genecards.org/cgi-bin/carddisp.pl?gene=MAPK14&search=MAPK14) | Mitogen-activated protein kinase 14 |
| [Q00535](https://www.uniprot.org/uniprotkb/Q00535/entry) | [CDK5](http://zinc15.docking.org/genes/CDK5) | Cyclin-dependent kinase 5 |
| [P11309](https://www.uniprot.org/uniprotkb/P11309/entry) | [PIM1](http://www.genecards.org/cgi-bin/carddisp.pl?gene=PIM1&search=PIM1) | Serine/threonine-protein kinase pim-1 |
| [Q02750](https://www.uniprot.org/uniprotkb/Q02750/entry) | [MAP2K1](http://www.genecards.org/cgi-bin/carddisp.pl?gene=MAP2K1&search=MAP2K1) | Dual specificity mitogen-activated protein kinase kinase 1 |
| [P42345](https://www.uniprot.org/uniprotkb/P42345/entry) | [MTOR](http://www.genecards.org/cgi-bin/carddisp.pl?gene=MTOR&search=MTOR) | Serine/threonine-protein kinase mTOR |
| [P43119](https://www.uniprot.org/uniprotkb/P43119/entry) | [PTGIR](http://www.genecards.org/cgi-bin/carddisp.pl?gene=PTGIR&search=PTGIR) | Prostacyclin receptor |
| [P28482](https://www.uniprot.org/uniprotkb/P28482/entry) | [MAPK1](http://www.genecards.org/cgi-bin/carddisp.pl?gene=MAPK1&search=MAPK1) | Mitogen-activated protein kinase 1 |
| [Q16539](https://www.uniprot.org/uniprotkb/Q16539/entry) | [MAPK14](http://www.genecards.org/cgi-bin/carddisp.pl?gene=MAPK1&search=MAPK1) | Mitogen-activated protein kinase 14 |
| [Q15722](https://www.uniprot.org/uniprotkb/Q15722/entry) | [LTB4R](http://www.genecards.org/cgi-bin/carddisp.pl?gene=LTB4R&search=LTB4R) | Leukotriene B4 receptor 1 |
| [Q8TDS5](https://www.uniprot.org/uniprotkb/Q8TDS5/entry) | [OXER1](http://www.genecards.org/cgi-bin/carddisp.pl?gene=OXER1&search=OXER1) | Oxoeicosanoid receptor 1 |
| [P32246](https://www.uniprot.org/uniprotkb/P32246/entry) | [CCR1](http://www.genecards.org/cgi-bin/carddisp.pl?gene=CCR1&search=CCR1) | C-C chemokine receptor type 1 |
| [P51677](https://www.uniprot.org/uniprotkb/P51677/entry) | [CCR3](http://www.genecards.org/cgi-bin/carddisp.pl?gene=CCR3&search=CCR3) | C-C chemokine receptor type 3 |
| [P53779](https://www.uniprot.org/uniprotkb/P53779/entry) | MAPK10 | Mitogen-activated protein kinase 10 |
| [Q5ECR9](https://www.uniprot.org/uniprotkb/Q5ECR9/entry) | [CCR5](http://www.genecards.org/cgi-bin/carddisp.pl?gene=CCR5&search=CCR5) | C-C chemokine receptor type 5 |
| [O14920](https://www.uniprot.org/uniprotkb/O14920/entry) | [IKBKB](http://www.genecards.org/cgi-bin/carddisp.pl?gene=IKBKB&search=IKBKB) | Inhibitor of nuclear factor kappa-B kinase subunit beta |
| [Q9NR96](https://www.uniprot.org/uniprotkb/Q9NR96/entry) | [TLR9](http://www.genecards.org/cgi-bin/carddisp.pl?gene=TLR9&search=TLR9) | Toll-like receptor 9 |
| [P51812](https://www.uniprot.org/uniprotkb/P51812/entry) | [RPS6KA3](http://www.genecards.org/cgi-bin/carddisp.pl?gene=RPS6KA3&search=RPS6KA3) | Ribosomal protein S6 kinase alpha-3 |
| [P49682](https://www.uniprot.org/uniprotkb/P49682/entry) | [CXCR3](http://www.genecards.org/cgi-bin/carddisp.pl?gene=CXCR3&search=CXCR3) | C-X-C chemokine receptor type 3 |

**Table S3: KEGG pathway analysis of potential target gene functions**

| **#term ID** | **term description** | **observed gene count** | **false discovery rate** | **matching proteins in the network** |
| --- | --- | --- | --- | --- |
| hsa04010 | MAPK signaling pathway | 16 | 2.94E-16 | MAPK1,FLT3,MAPK3,EGFR,DUSP16,MAP2K1,PRKACA,CHUK,RPS6KA3,MAPK8,MAPK9,TNF,IKBKB,AKT1,IRAK4,CDC42 |
| hsa04657 | IL-17 signaling pathway | 13 | 3.26E-15 | MAPK1,MMP13,MAPK3,MMP3,MMP1,PTGS2,CHUK,MMP9,MAPK8,IL6,MAPK9,TNF,IKBKB |
| hsa04933 | JAK-STAT signaling pathway | 13 | 5.70E-15 | MAPK1,MMP2,SERPINE1,CDKN1B,MAPK3,PIM1,JAK2,MAPK8,IL6,MAPK9,TNF,AKT1,CDC42 |
| hsa04151 | PI3K-Akt signaling pathway | 18 | 3.39E-14 | MAPK1,RPS6KB1,CDKN1B,FLT3,MAPK3,CDK2,EGFR,MAP2K1,MTOR,CHUK,TLR4,SYK,JAK2,IL6,IKBKB,JAK3,AKT1,JAK1 |
| hsa04660 | NF-kappa B signaling pathway | 13 | 7.61E-15 | MAPK1,MAPK3,MAP2K1,FYN,CHUK,MAPK8,MAPK9,TNF,PTPRC,IKBKB,AKT1,LCK,CDC42 |
| hsa01521 | EGFR tyrosine kinase inhibitor resistance | 11 | 3.27E-13 | MAPK1,RPS6KB1,MAPK3,EGFR,MAP2K1,MTOR,SRC,JAK2,IL6,AKT1,JAK1 |
| hsa05167 | Kaposi sarcoma-associated herpesvirus infection | 16 | 2.15E-12 | MAPK1,EIF2AK2,MAPK3,CCR5,CCR1,MAP2K1,MTOR,PTGS2,CHUK,SRC,SYK,HCK,JAK2,MAPK8,IL6,MAPK9,IKBKB,CCR3,AKT1,JAK1 |
| hsa04062 | Chemokine signaling pathway | 13 | 3.21E-12 | EIF2AK2,TLR9,MTOR,CHUK,SRC,SYK,JAK2,IL6,TNF,IKBKB,AKT1,IRAK4,JAK1 |
| hsa05163 | Human cytomegalovirus infection | 19 | 1.95E-14 | MAPK1,RPS6KB1,MAPK3,EGFR,CCR5,CCR1,MAP2K1,PRKACA,MTOR,PTGS2,CHUK,SRC,PTK2B,IL6,TNF,IKBKB,CCR3,AKT1,JAK1 |
| hsa05203 | Viral carcinogenesis | 18 | 2.59E-14 | HDAC7,MAPK1,CDKN1B,EIF2AK2,MAPK3,CDK2,CCR5,HDAC3,PRKACA,HDAC8,SRC,SYK,CDK1,CHEK1,JAK3,CCR3,CDC42,JAK1 |
| hsa05168 | Herpes simplex virus 1 infection | 13 | 8.21E-14 | MAPK1,MAPK3,CCR5,CCR1,MAP2K1,PRKACA,CHUK,SRC,CXCR3,HCK,JAK2,PTK2B,IKBKB,JAK3,CCR3,AKT1,CDC42 |
| hsa05170 | Human immunodeficiency virus 1 infection | 17 | 2.30E-12 | MAPK1,RPS6KB1,MAPK3,CCR5,MAP2K1,MTOR,CHUK,TLR4,CDK1,MAPK8,PTK2B,CHEK1,MAPK9,TNF,IKBKB,AKT1,IRAK4 |
| hsa05166 | Human T-cell leukemia virus 1 infection | 16 | 6.16E-15 | MAPK1,MAPK3,CDK2,MAP2K1,PRKACA,CHUK,MAPK8,IL6,CHEK1,MAPK9,TNF,IKBKB,JAK3,AKT1,LCK,JAK1 |
| hsa04620 | Toll-like receptor signaling pathway | 13 | 7.61E-15 | MAPK1,MAPK3,MAP2K1,TLR9,CHUK,TLR4,MAPK8,IL6,MAPK9,TNF,IKBKB,AKT1,IRAK4 |
| hsa04668 | TNF signaling pathway | 13 | 2.24E-14 | MAPK1,MAPK3,MMP3,MAP2K1,PTGS2,CHUK,MMP9,MAPK8,IL6,MAPK9,TNF,IKBKB,AKT1 |
| hsa05165 | Human papillomavirus infection | 17 | 1.25E-13 | MAPK1,RPS6KB1,CDKN1B,EIF2AK2,MAPK3,CDK2,EGFR,MAP2K1,PRKACA,MTOR,PTGS2,CHUK,TNF,IKBKB,AKT1,CDC42,JAK1 |
| hsa05169 | Epstein-Barr virus infection | 14 | 4.37E-13 | CDKN1B,EIF2AK2,CDK2,CHUK,SYK,MAPK8,IL6,MAPK9,TNF,IKBKB,JAK3,AKT1,IRAK4,JAK1 |
| hsa05164 | Influenza A | 13 | 1.17E-12 | MAPK1,EIF2AK2,MAPK3,MAP2K1,CHUK,TLR4,JAK2,IL6,TNF,IKBKB,AKT1,IRAK4,JAK1 |
| hsa04664 | Fc epsilon RI signaling pathway | 10 | 2.17E-12 | MAPK1,MAPK3,MAP2K1,FYN,ALOX5,SYK,MAPK8,MAPK9,TNF,AKT1 |
| hsa04066 | HIF-1 signaling pathway | 11 | 4.33E-12 | MAPK1,SERPINE1,RPS6KB1,CDKN1B,MAPK3,EGFR,MAP2K1,MTOR,TLR4,IL6,AKT1 |
| hsa04658 | Th1 and Th2 cell differentiation | 10 | 2.26E-11 | MAPK1,MAPK3,CHUK,JAK2,MAPK8,MAPK9,IKBKB,JAK3,LCK,JAK1 |
| hsa04014 | Ras signaling pathway | 12 | 7.18E-10 | MAPK1,FLT3,MAPK3,EGFR,MAP2K1,PRKACA,CHUK,MAPK8,MAPK9,IKBKB,AKT1,CDC42 |
| hsa04666 | Fc gamma R-mediated phagocytosis | 9 | 9.51E-10 | MAPK1,RPS6KB1,MAPK3,MAP2K1,SYK,HCK,PTPRC,AKT1,CDC42 |
| hsa04064 | T cell receptor signaling pathway | 9 | 2.29E-09 | PARP1,PTGS2,CHUK,TLR4,SYK,TNF,IKBKB,LCK,IRAK4 |
| hsa04150 | mTOR signaling pathway | 10 | 3.11E-09 | MAPK1,RPS6KB1,MAPK3,MAP2K1,MTOR,CHUK,RPS6KA3,TNF,IKBKB,AKT1 |
| hsa04370 | VEGF signaling pathway | 7 | 2.09E-08 | MAPK1,MAPK3,MAP2K1,PTGS2,SRC,AKT1,CDC42 |
| hsa04510 | Focal adhesion | 10 | 2.87E-08 | MAPK1,MAPK3,EGFR,MAP2K1,FYN,SRC,MAPK8,MAPK9,AKT1,CDC42 |
| hsa04024 | cAMP signaling pathway | 7 | 5.49E-05 | MAPK1,MAPK3,MAP2K1,PRKACA,MAPK8,MAPK9,AKT1 |

**Table S4: Mode of action for *P. harmala*-CS-ZnO NPs formula against Herpes Simplex Virus Type 1 (HSV-1)**

| **Mechanism** | **Conc. µg/ml** | **Virus Control (PFU/ml)** | **Viral Count Post- Treatment (PFU/ml)** | **Viral Inhibition %** |
| --- | --- | --- | --- | --- |
| **Virucidal** | **50** | **1.9 * 10^6^** | **1.3 * 10^6^** | **31.5%** |
|  | **25** |  | **1.4* 10^6^** | **26%** |
|  | **12.5** |  | **1.53 * 10^6^** | **19.5%** |
| **Viral Adsorption** | **50** | **1.9 * 10^6^** | **1.6 * 10^6^** | **16%** |
|  | **25** |  | **1.61 * 10^6^** | **15.2%** |
|  | **12.5** |  | **1.66 * 10^6^** | **12.6%** |
| **Viral Replication** | **50** | **1.9 * 10^6^** | **1.76 * 10^6^** | **7.4%** |
|  | **25** |  | **1.78 * 10^6^** | **6%** |
|  | **12.5** |  | **1.8 * 10^6^** | **5%** |

**a**

**
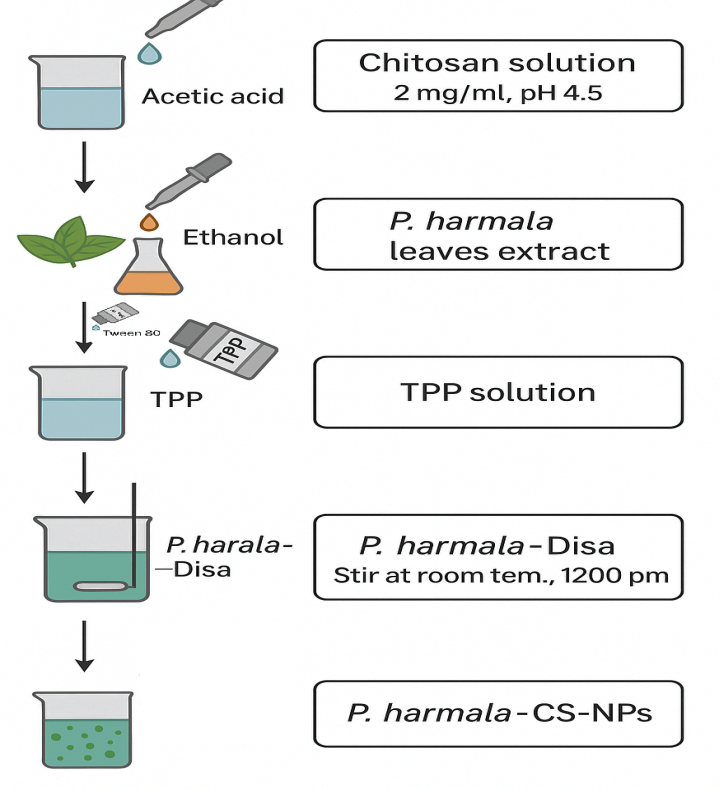

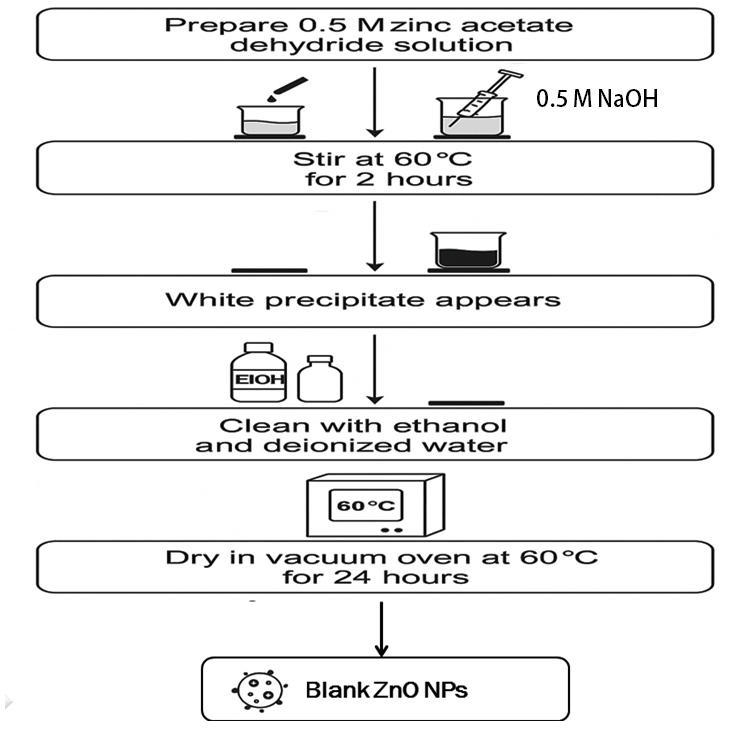

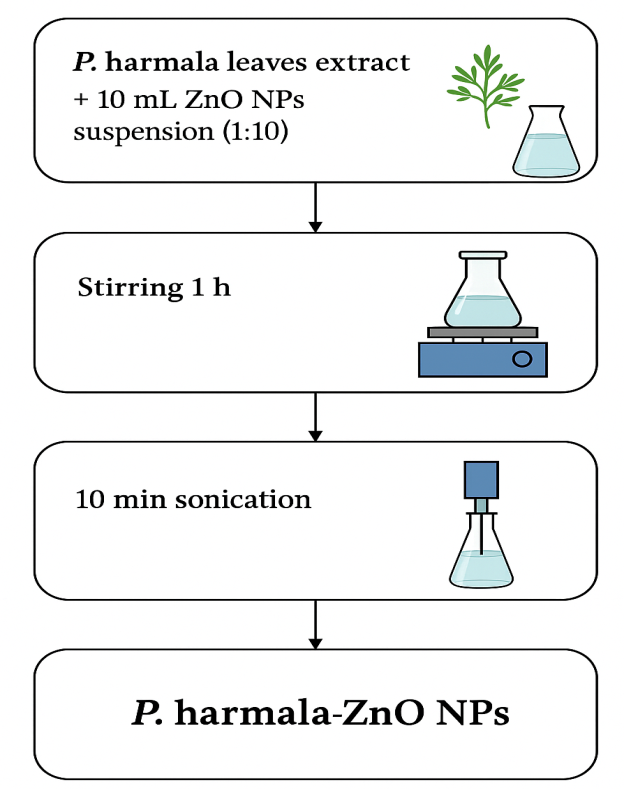
**

**b**

**c**

**d**

**e**

**
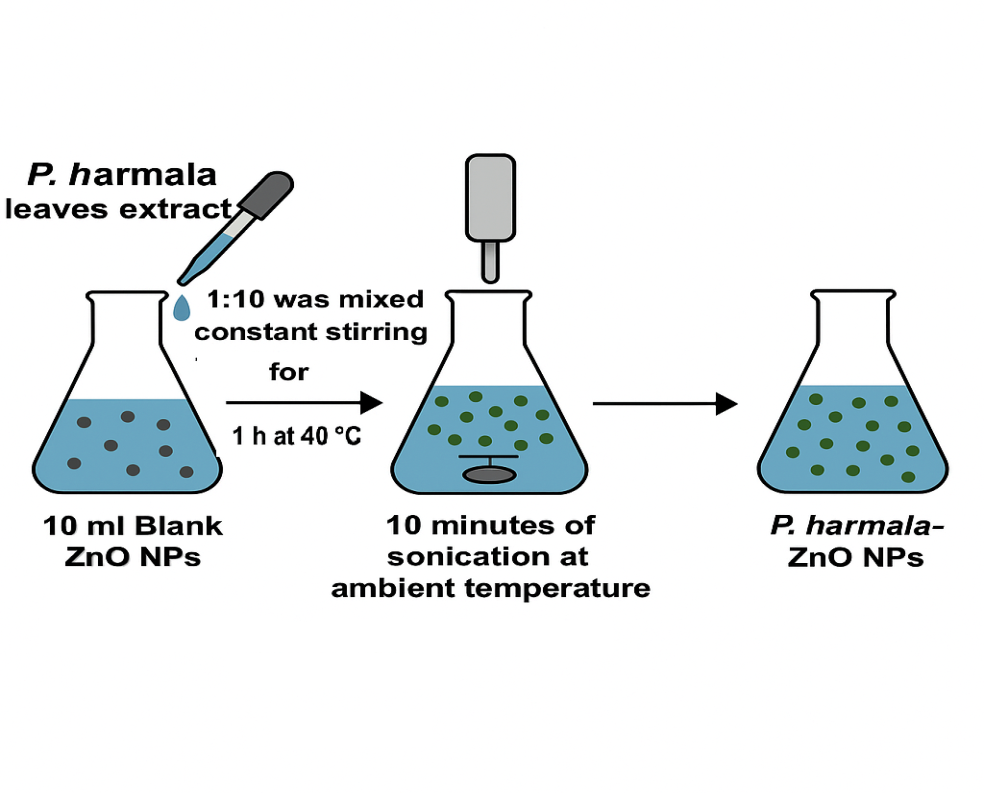

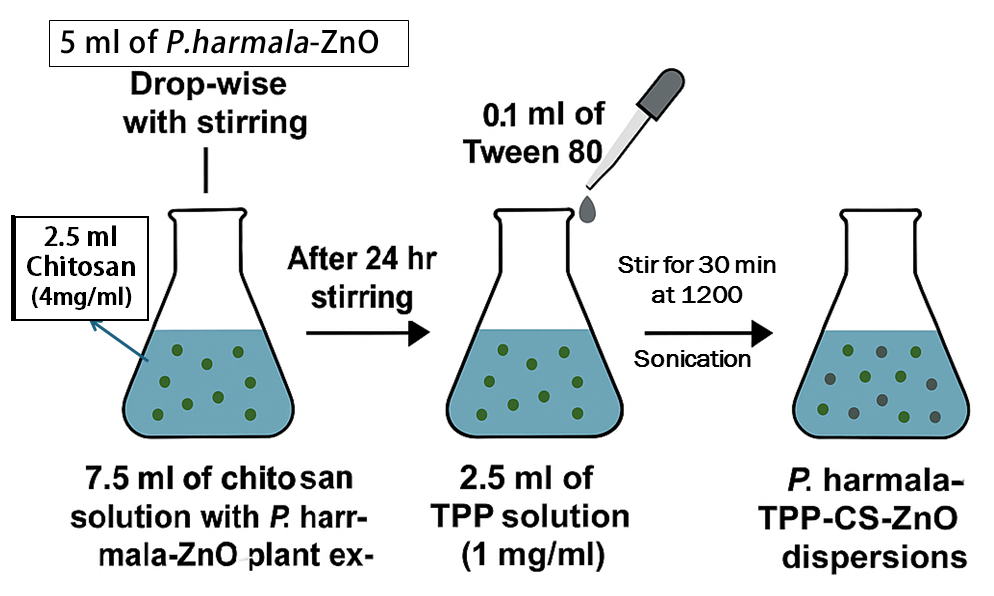
**

**Fig. S1: Schematic representation of the preparation of** **(a); *P. harmala*-CS-NPs and (b);** **Blank ZnO NPs and (c); *P. harmala*- ZnO NPs and (d); *P. harmala* -CS-ZnO NPs and (e); *P. harmala* - TPP-CS-ZnO NPs**


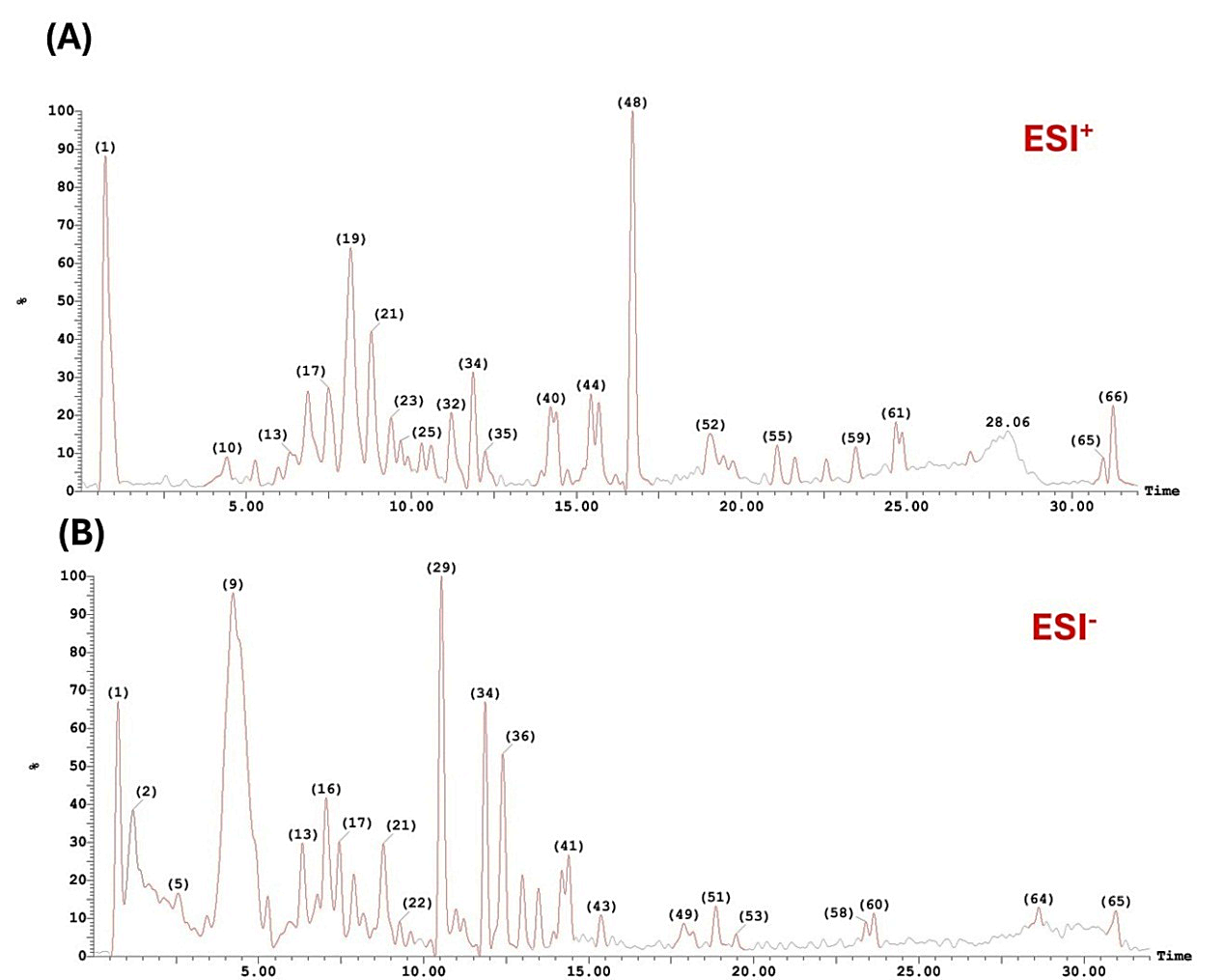


**Fig. S2: Base peak chromatograms (BPCs) of *P. harmala* leaves extract in both positive and negative ionization modes.**

**
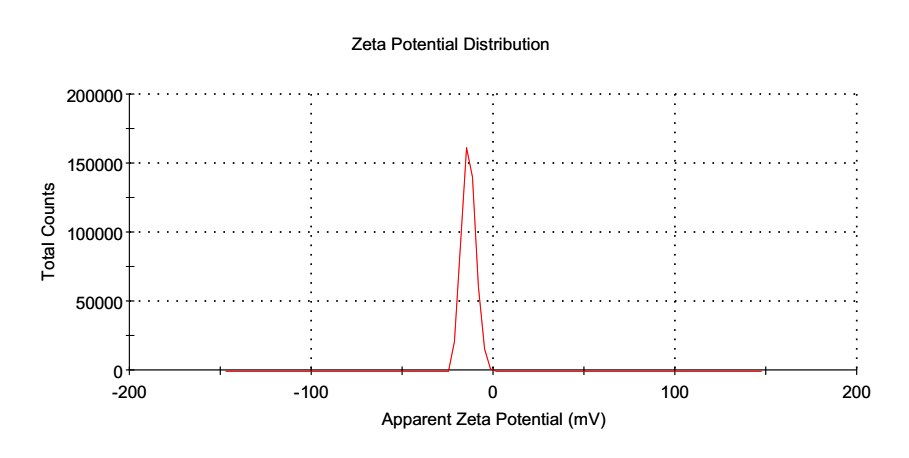

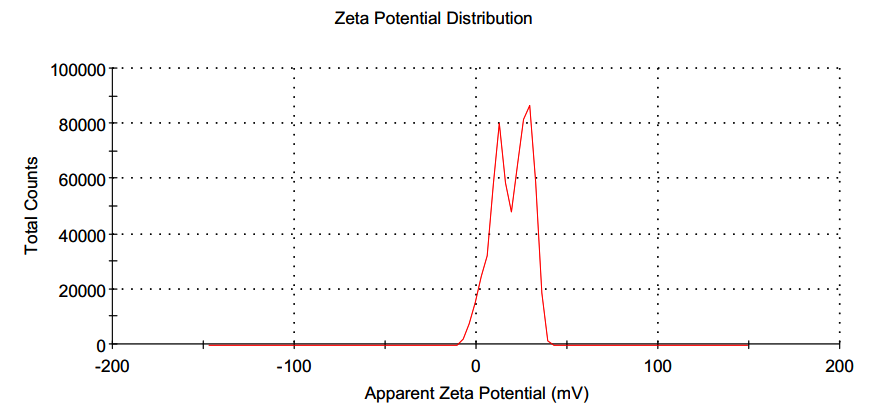
**

**(b)**

**(a)**

**
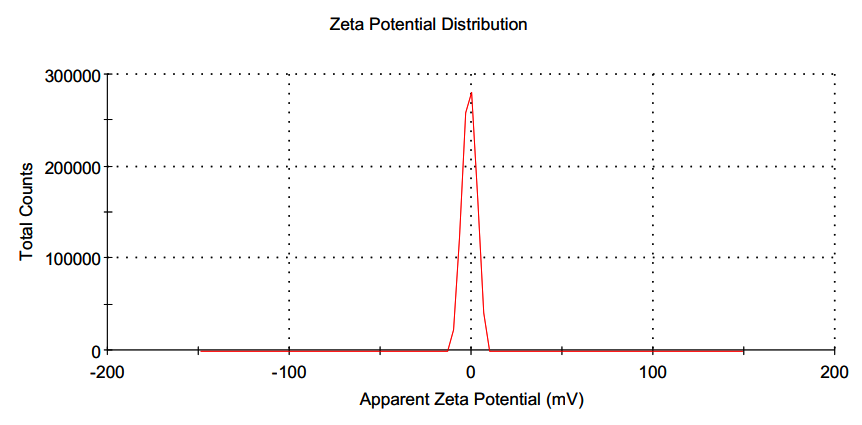

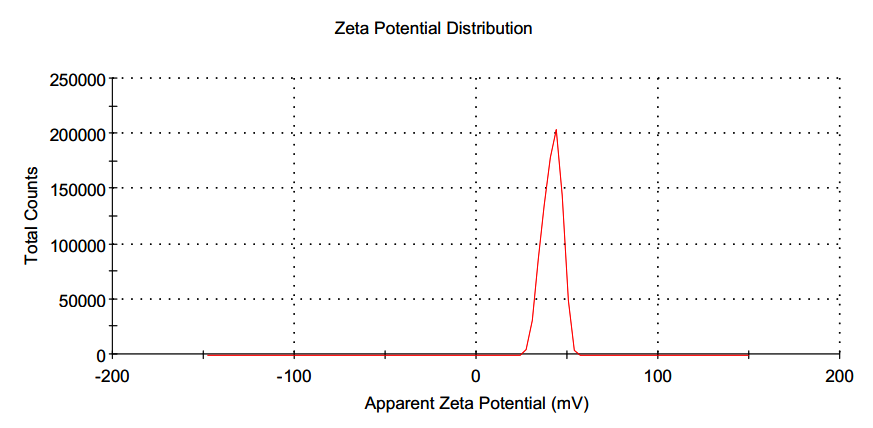
**

**(d)**

**(c)**

**
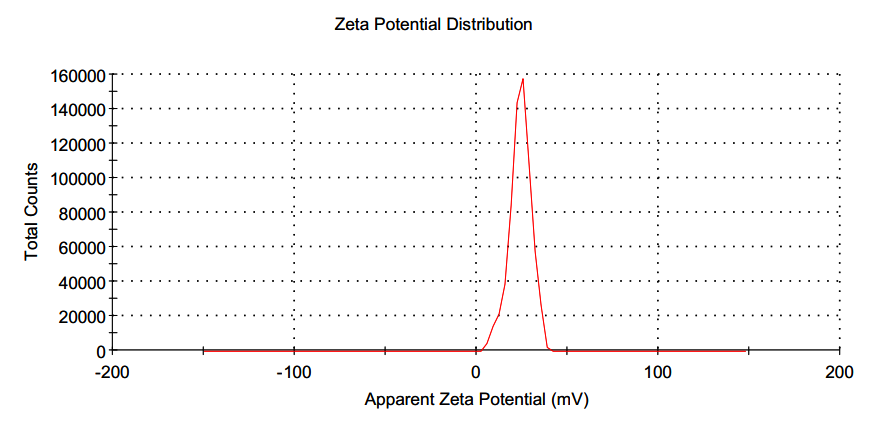
**

**(e)**

**Fig. S3: Zeta potential of of (a); Blank ZnO NPs and (b); *P. harmala*-CS-NPs and (c); *P. harmala*- ZnO NPs and (d); *P. harmala* -CS-ZnO NPs and (e); *P. harmala* - TPP-CS-ZnO NPs.**

**
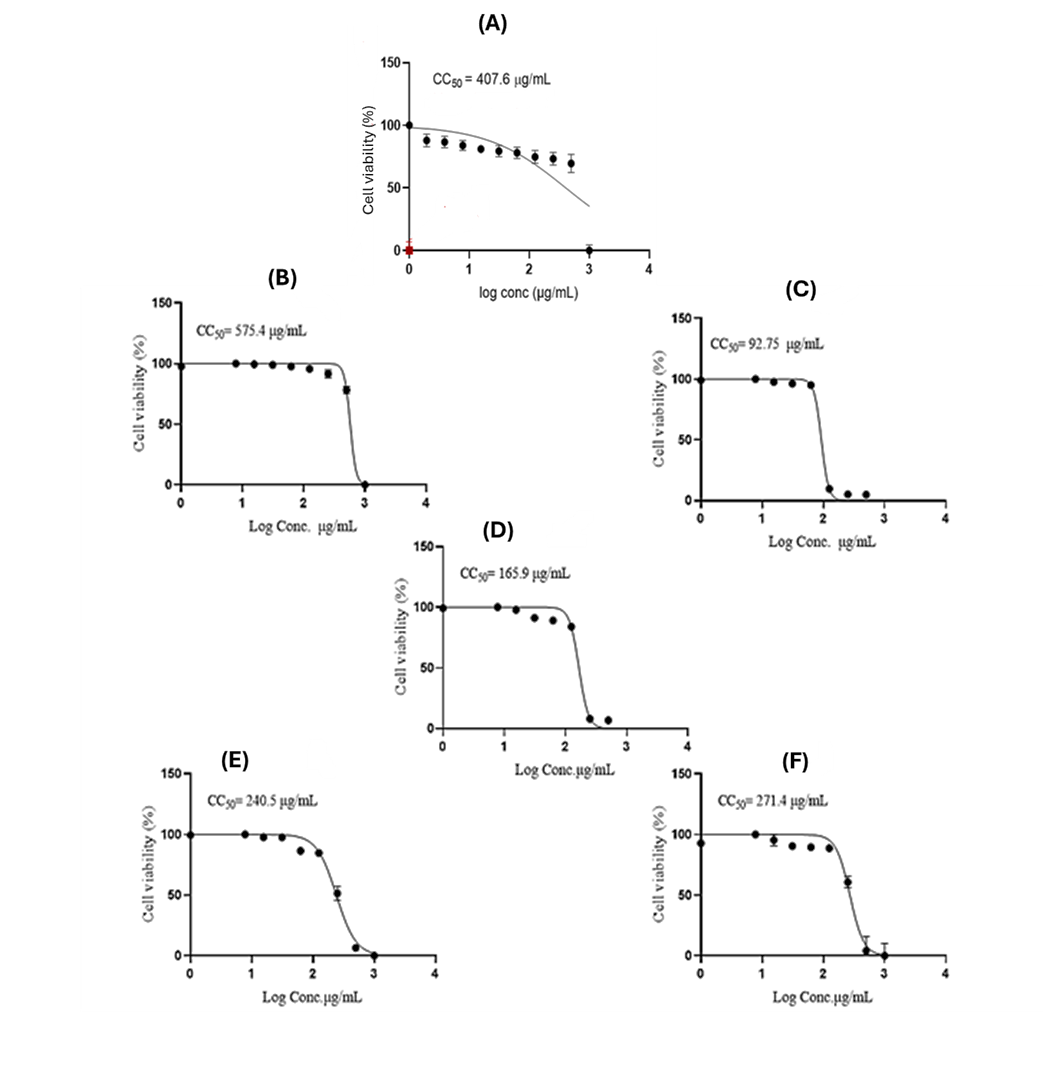
**

**Fig. S4: Cytotoxicity graphs with CC_50_ values of *P. harmala* leaves extract and its nano-formulations. (A) *P. harmala* leaves extract (B) *P. harmala*-CS-NPs, (C) Blank ZnO NPs*, (D), P. harmala*-ZnO NPs (E) *P. harmala*-TPP-CS-ZnO NPs and (F) *P. harmala*-CS-ZnO NPs.**
